# New Perspectives on the Efficacy of Governor Vessel Moxibustion Combined With Rehabilitation Training for Poststroke Muscle Spasticity: A Systematic Review and Meta‐Analysis of Randomized Controlled Trials

**DOI:** 10.1002/brb3.71346

**Published:** 2026-03-25

**Authors:** Jun‐Xiang Wang, Zhe‐Hao Hu, Yi‐Yang Song, Jin‐Shan Zhong, Miao He

**Affiliations:** ^1^ School of Nursing Beijing University of Chinese Medicine Beijing China

**Keywords:** meta‐analysis, motor dysfunction, moxibustion, muscle spasticity, stroke rehabilitation

## Abstract

**Background:**

Muscle spasticity, a prevalent motor impairment after stroke, substantially diminishes patients' quality of life. Governor Vessel moxibustion (GVM), a significant complementary therapy, shows promise, yet the current evidence remains inadequate. This study aimed to evaluate the efficacy of GVM with routine rehabilitation training in alleviating poststroke spasticity (PSS).

**Methods:**

We searched PubMed, Cochrane Library, Embase, Web of Science, four Chinese databases, and clinical trials registry for randomized controlled trials (RCTs) of PSS patients who mainly received GVM treatment alongside conventional rehabilitation. Statistical evaluations were conducted using Review Manager 5.4 and R Studio. For assessing bias in the included studies, the Cochrane risk of bias tool (RoB 2.0) was used to evaluate RCTs.

**Results:**

Eleven studies involving 859 PSS patients were included. Meta‐analysis demonstrated that when combined with routine rehabilitation training, GVM effectively alleviated muscle spasticity, as measured by the modified Ashworth scale (MD, −0.65 [95% CI, −0.84 to −0.47], *p* < 0.01) and the composite spasticity index (MD, −1.82 [95% CI, −2.25 to −1.39], *p* < 0.01). The treatment time and frequency could potentially be the main contributors to heterogeneity. Results suggested that a treatment regimen consisting of 60‐min sessions conducted once‐weekly and lasting for at least 8 weeks was the most effective, providing evidence for optimizing clinical application of GVM combined with rehabilitation training in PSS management.

**Conclusion:**

GVM serves as an effective complementary therapy to routine rehabilitation for PSS patients, with generally mild adverse events. However, before formulating evidence‐based and definitive recommendations, more rigorously designed and in‐depth research is warranted.

## Graphical abstract

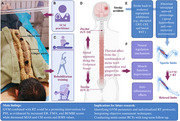

## 1 Introduction

Stroke, a prevalent neurological disorder, is marked by high mortality, recurrence, and disability rates (Ozkan et al. 2025). Over the past three decades, the global burden of stroke has risen substantially, particularly in lower‐income and lower‐middle‐income countries, with approximately 70%–80% of patients experiencing varying degrees of functional impairment (Li et al. 2021; Brusola et al. 2023; Suputtitada et al. 2024). Among stroke survivors, nearly one‐third develop poststroke spasticity (PSS), a neuromuscular disorder characterized by involuntary muscle contractions, increased muscle tone, and restricted joint mobility (Persson et al. 2020). These symptoms lead to pain, joint deformities, impaired motor function, and reduced ability to perform daily activities, severely diminishing quality of life (Suputtitada et al. 2024). Therefore, effective management of PSS has become one of the hot topics in modern medical research.

Currently, a well‐defined and standardized protocol for PSS management remains unestablished. Modern interventions are selected progressively based on spasticity severity: focal treatments (e.g., phenol/alcohol neurolysis plus botulinum toxin injections) for localized cases (Marsden et al. 2023), systemic oral medications for mild‐to‐moderate generalized spasticity (Alshahrani 2023), and invasive options (e.g., intrathecal baclofen and surgery) for refractory severe cases (Kudva et al. 2021). Importantly, rehabilitation training (RT) is an integral, concurrent component throughout PSS management—early intervention (within 1 month poststroke) is critical for functional recovery, regardless of other modern treatments used (Coleman et al. 2017). Nevertheless, conventional methods have limitations; medications may cause muscle weakness or dizziness, invasive procedures carry risks, and RT often requires a prolonged course (Alshahrani 2023). Thus, complementary therapies (e.g., moxibustion and acupuncture) show promise as adjuncts, helping alleviate medication side effects and shorten RT duration to address these gaps.

**FIGURE 1:**
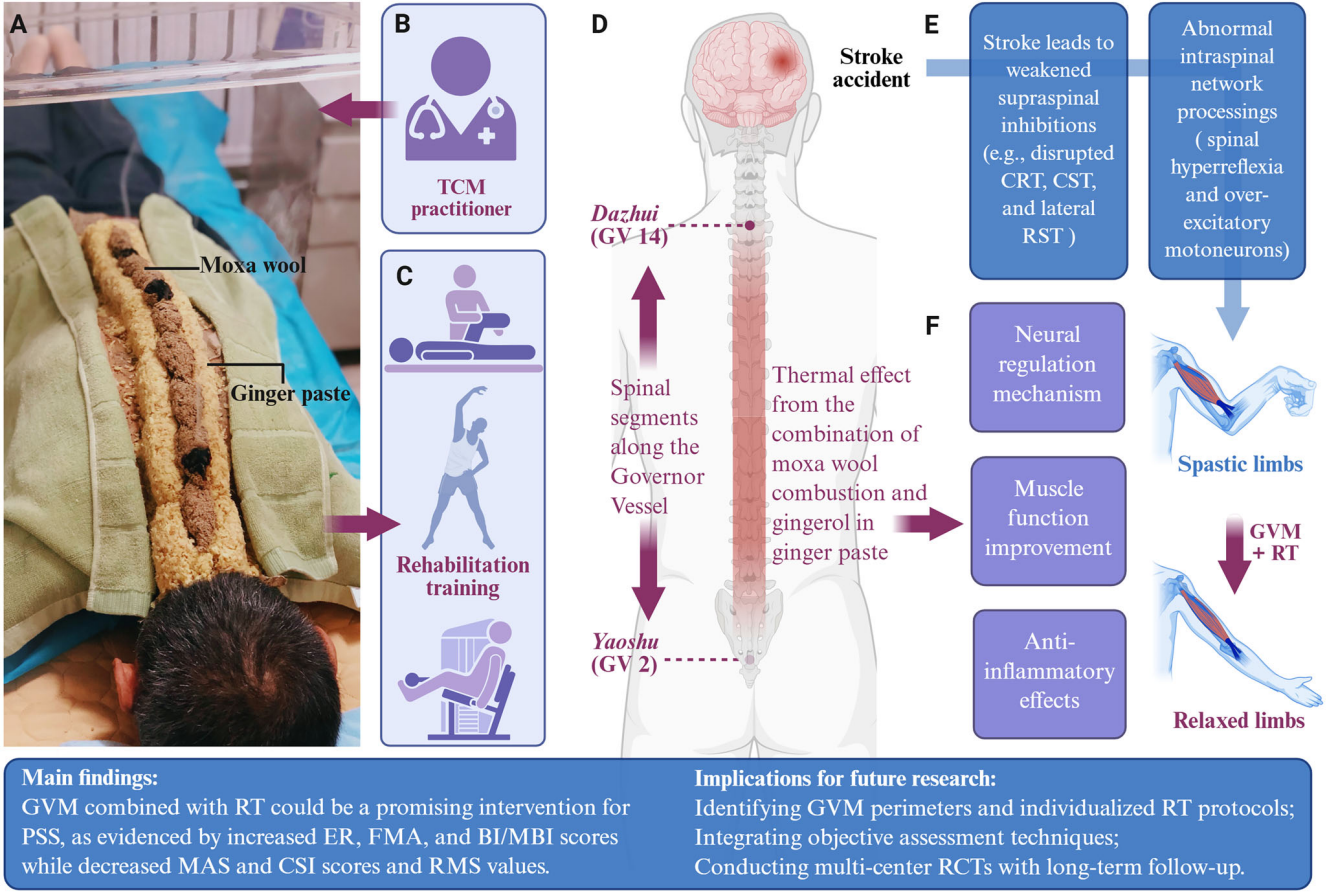
Visual summary. (A) Picture of GVM treatment, (B) roles of TCM practitioner, (C) RT at different stages from acute to chronic stroke, (D) schematic diagram of GVM, (E) pathogenesis of PSS, and (F) possible antispastic mechanisms of GVM. Abbreviations: BI/MBI, Barthel Index/modified Barthel Index; CRT, cortico‐reticular tract; CSI, composite spasticity index; CST, corticospinal tract; EMG, electromyography; ER, effective rate; FMA, Fugl–Myer assessment; GVM, Governor Vessel moxibustion; MAS, modified Ashworth scale; PSS, poststroke spasticity; RMS, root mean square value; RST, reticulospinal tract; RT, rehabilitation training; TCM, traditional Chinese medicine.

Within this context, moxibustion is increasingly integrated into RT for PSS, showing considerable therapeutic promise (Wei et al. 2016). Governor Vessel moxibustion (GVM), a widely used moxibustion technique, involves indirect application (i.e., ginger‐partitioned moxibustion) along the Governor Vessel (GV)—specifically over spinal segments from Dazhui (GV14) to Yaoshu (GV2) (Figure 1A,D) (Zhang et al. 2017). Characterized by a large treatment area, thick moxa cones, and intense thermal effect, GVM combines physical heat stimulation with the pharmacological properties of moxa wool and ginger (Zhang et al. 2017). In traditional Chinese medicine (TCM), these attributes are believed to dredge meridians, invigorate qi, and promote blood circulation—addressing core pathogenic factors underlying PSS, such as qi stagnation and blood stasis (Liao et al. 2023).

As a cornerstone of PSS management, RT enhances motor function through repetitive training that facilitates neural plasticity (Coleman et al. 2017). Notably, combining GVM with RT may yield synergistic benefits: GVM modulates central nervous system regulation via its thermal and pharmacological actions, while RT reinforces these gains by consolidating functional recovery through motor learning. Growing clinical data suggest that adding GVM to conventional RT yields superior antispastic effects compared to RT alone (Feng et al. 2014; Wang et al. 2017; Huang and Zhong 2018; Wang and Sun 2021; Wu et al. 2022; Ding et al. 2022; Chen et al. 2023; Zhao et al. 2023; Sun and Dong 2023; Tong et al. 2023; Xu and Qin 2024). However, current evidence remains inconclusive, highlighting the need for this systematic review.

## 2 Aim

The aim of this systematic review and meta‐analysis was to comprehensively evaluate the efficacy and safety of GVM as a crucial complementary therapy for PSS, and to synthesize all available evidence thoroughly to support its clinical application.

## 3 Methods

### 3.1 Protocol and Registration

For this research, we adhered to the recommendations of the *Cochrane Handbook for Systematic Reviews of Interventions* (Higgins et al. 2025) and the Preferred Reporting Items for Systematic Reviews and Meta‐Analyses (PRISMA) statement (Moher et al. 2015). The protocol had been previously registered at PROSPERO (ID: CRD42024519063).

### 3.2 Inclusion and Exclusion Criteria

The Participant, Intervention, Comparison, Outcome, and Study Design (PICOS) framework was adopted to formulate the inclusion criteria:

P (Participants): Participants aged 18 years or older, diagnosed with PSS according to explicit diagnostic criteria or references, were included. There were no restrictions regarding gender, disease duration, or disease severity.

I (Interventions): The intervention group received GVM as the core treatment, which could be combined with routine RT; additional adjunctive therapies (e.g., acupuncture and acupressure) were allowed only if they were consistent with the control group. The original literature must clearly describe the GVM procedure, including details of disinfection, treatment time, frequency, course, and posttreatment protocols.

C (Controls): The control group received the same routine RT and any other adjunctive therapies as the intervention group, excluding GVM.

O (Outcomes): The primary outcome was the spasticity degree, assessed by two of the most prevalently used clinical scales: the modified Ashworth scale (MAS) and the composite spasticity index (CSI). Secondary outcomes included the effective rate (ER), root mean square (RMS) values of the biceps brachii, Fugl–Meyer assessment (FMA) score, Berg Balance scale (BBS) score, Barthel Index (BI)/modified Barthel Index (MBI) score, and adverse events (AE).

S (Study types): Randomized controlled trials (RCTs).

The exclusion criteria were as follows: (1) studies lacking definite diagnostic criteria or those where muscle spasticity was caused by factors other than stroke, (2) investigations with incomplete outcome data, and (3) duplicate publications.

### 3.3 Search Strategies

We conducted a comprehensive search across multiple databases, including PubMed, the Cochrane Library, Embase, Web of Science, Chinese Biomedical Literature Database (CBMdisc), China National Knowledge Infrastructure (CNKI), Chinese Scientific Journal Database (VIP), and Wanfang Database. Meanwhile, we conducted a manual search of the Chinese Clinical Trials Registry and the US Health Ongoing Trials Registry (www.ClinicalTrials.gov) to identify potentially eligible trials. The search spanned from the inception date of each database up to January 1, 2025. The search terms mainly consisted of “moxibustion”, “stroke”, and “spasticity”. Two reviewers (Z.‐H. Hu and Y.‐Y. Song) independently carried out the screening of article titles and abstracts using EndNote 21. Subsequent to the abstract screening, the full‐text articles were downloaded for in‐depth perusal. In case of any discrepancies between the two reviewers, an initial discussion was held. If the issue remained unresolved, the corresponding author of the relevant article would be consulted for clarification.

### 3.4 Data Extraction and Quality Assessment

Two reviewers utilized a standardized data table for information extraction. In cases where disagreements arose, a third reviewer (J.‐X. Wang) was called upon to make an assessment. If the necessary information was either incomplete or unclear, the corresponding author was contacted via email. The revised Cochrane risk of bias tool for RCTs, RoB 2.0 (Cumpston et al. 2019), was employed to assess the risk of bias in the included studies. This tool assesses bias risks across five domains: (1) bias in the randomization process, (2) bias in deviations from intended interventions, (3) bias in missing outcome data, (4) bias in outcome measurement, and (5) bias in the selection of the reported results. For each domain and in the overall judgment, each RCT was assigned one of three ratings: “low risk”, “some concerns,” or “high risk”. Two reviewers independently used it to determine the risk of bias for each included trial (Higgins et al. 2025). Any disagreements were resolved through consensus.

### 3.5 Data Analysis

Review Manager 5.4 and R Studio were used for data analysis. For primary and secondary outcomes excluding AE, results were presented as relative risk (RR) with a 95% confidence interval (CI). The effect estimates were calculated based on the post‐intervention values. Statistical analysis was carried out in accordance with the statistical guidelines specified in the latest *Cochrane Handbook for Systematic Reviews of Interventions* (Higgins et al. 2025). Meta‐analyses were performed when the trials demonstrated good homogeneity in study design, participants, intervention, control, and outcomes. When *I^2^* ≤ 50% and *p* ≥ 0.1, the fixed‐effects model was performed for meta‐analyses; otherwise, a random‐effects model was used. If there was significant heterogeneity between studies (*I^2^* > 50%), the source of heterogeneity would be analyzed. Subgroup analysis was conducted when different types of controls were used.

## 4 Results

### 4.1 Study Selection

**FIGURE 2:**
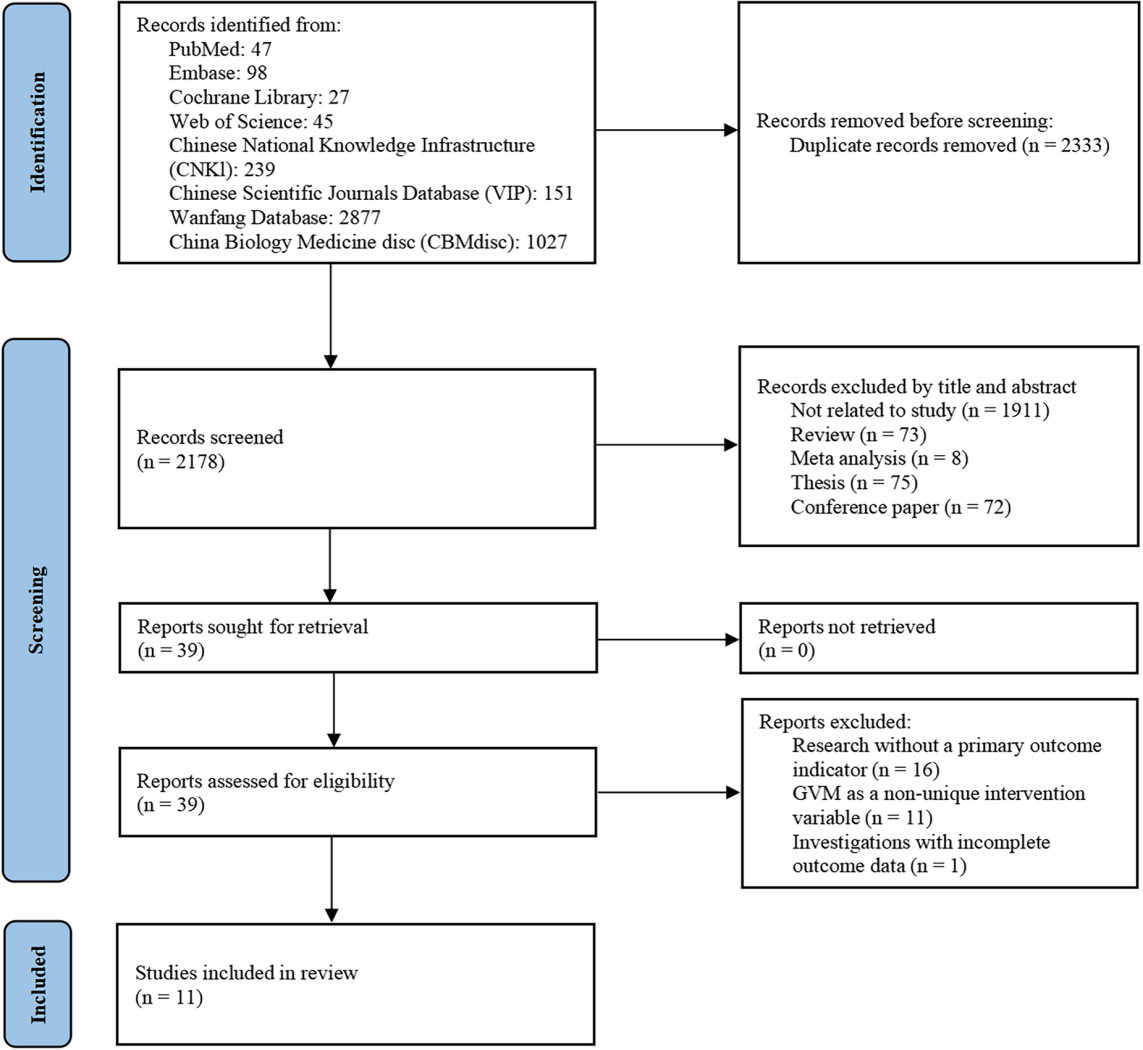
Flow diagram of literature screening.

A total of 4511 literatures were initially retrieved. Subsequently, through screening of titles and abstracts, only 39 remained. The full text of these 39 literatures was perused. Eventually, after excluding 28 literatures, 11 literatures were enrolled (Feng et al. 2014; Wang et al. 2017; Huang and Zhong 2018; Wang and Sun 2021; Wu et al. 2022; Ding et al. 2022; Chen et al. 2023; Zhao et al. 2023; Sun and Dong 2023; Tong et al. 2023; Xu and Qin 2024). The screening process is shown in Figure 2.

### 4.2 Study Characteristics

Eleven RCTs involving a total of 859 participants (430 in the treatment group and 429 in the control group) mainly integrated GVM and RT. Among these, seven trials (Feng et al. 2014; Wang et al. 2017; Wu et al. 2022; Ding et al. 2022; Chen et al. 2023; Tong et al. 2023; Xu and Qin 2024) directly compared GVM combined RT versus RT. The remaining four (Huang and Zhong 2018; Wang and Sun 2021; Zhao et al. 2023; Sun and Dong 2023) incorporated acupuncture/acupressure, with GVM being the only variable factor under study. The treatment duration across these trials ranged from 4 to 9 weeks. The mean age of participants in the intervention group was 55.36 ± 13.88 years, compared with 55.11 ± 13.97 years in the control group. The stroke types encompassed ischemic stroke, hemorrhagic stroke, and unspecified stroke types. All the trials were conducted in China. While one trial was published in English, the rest were in Chinese. In terms of gender distribution, there were 474 male and 385 female participants. The detailed characteristics of the included studies are shown in Table 1.

**TABLE 1:** The characteristics of included studies.

Study ID	Year	Sample size (male/female)		Stroke types	Age of patients (year)		Outcomes	Intervention protocols		Treatment time	Treatment frequency	Treatment duration	Treatment area
		E	C		E	C		E	C	(min)		(weeks)	
**GVM + RT versus RT**													
Xu W. T.	2024 (Xu and Qin 2024)	46 (25/21)	46 (27/19)	Ischemic and hemorrhagic stroke	47–72 (63.05 ± 7.28)	49–75 (62.65 ± 7.41)	(1)	GVM + RT	RT	90	Six times a week	4	Lower limbs
Tong Q. S.	2023 (Tong et al. 2023)	30 (15/15)	29 (14/15)	Ischemic and hemorrhagic stroke	Unclear (60.47 ± 13.58)	Unclear (59.94 ± 11.32)	(1) (3) (5) (6)	GVM + RT	RT	90	Three times a week	6	Upper limbs
Chen S. Q.	2023 (Chen et al. 2023)	35 (18/17)	35 (19/16)	Unclear	42–74 (52.91 ± 6.24)	40–75 (53.69 ± 5.17)	(1) (3) (4) (5) (6) (7)	GVM + RT	RT	30	Five times a week	9	Upper limbs
Wu H. L.	2022 (Wu et al. 2022)	37 (23/14)	37 (22/15)	Ischemic and hemorrhagic stroke	42–70 (55.98 ± 1.32)	41–68 (56.01 ± 1.19)	(2) (5) (6)	GVM + RT	RT	90	Once a month	8	Lower limbs
Ding C. M.	2022 (Ding et al. 2022)	45 (24/21)	45 (21/24)	Ischemic and hemorrhagic stroke	55–67 (58.12 ± 9.14)	53–69 (57.32 ± 8.99)	(1) (4) (5) (6)	GVM + RT	RT	60	Once a week	6	Upper limbs
Wang Y. H.	2017 (Wang et al. 2017)	40 (27/13)	40 (24/16)	Ischemic and hemorrhagic stroke	41–70 (45.8 ± 13.4)	40–70 (46.5 ± 15.3)	(2) (5) (6)	GVM + RT	RT	90	Once a month	8	Lower limbs
Feng X. D.	2014 (Feng et al. 2014)	20 (14/6)	20 (13/7)	Ischemic and hemorrhagic stroke	Unclear (44.90 ± 9.86)	Unclear (44.35 ± 10.18)	(1) (4) (5) (6)	GVM + RT	RT	60	Once a week	8	Four limbs
**GVM + RT + Acu versus RT + Acu**													
Sun C. X.	2023 (Sun and Dong 2023)	40 (21/19)	40 (20/20)	Ischemic and hemorrhagic stroke	48–79 (44.6 ± 25.4)	49–78 (43.8 ± 26.1)	(1) (3) (5) (6)	GVM + RT + Acu	RT + Acu	Unclear	Five times a week	4	Upper limbs
Zhao H. L.	2023 (Zhao et al. 2023)	32 (17/15)	32 (17/15)	Unclear	Unclear	Unclear	(1) (6)	GVM + RT + Acu	RT + Acu	20	Three times a week	4	Four limbs
Wang Y. C.	2021 (Wang and Sun 2021)	50 (28/22)	50 (26/24)	Ischemic stroke	Unclear (67.6 ± 11.4)	Unclear (66.8 ± 11.9)	(1) (3) (4) (5) (6)	GVM + RT + Acu	RT + Acu	90	Once a week	4	Upper limbs
Huang Q. X.	2018 (Huang and Zhong 2018)	55 (29/26)	55 (30/25)	Unclear	35–71 (52.48 ± 4.01)	34–72 (52.50 ± 3.98)	(1) (2) (5) (6)	GVM + RT + Acu	RT + Acu	30	Unclear	6	Four limbs

*Note*: (1) modified Ashworth scale (MAS), (2) composite spasticity index (CSI), (3) effective rate (ER), (4) root mean square (RMS) value of surface electromyography (sEMG), (5) Fugl–Myer assessment (FMA), (6) Barthel Index (BI)/modified Barthel Index (MBI), and (7) adverse events (AE).

Abbreviations: Acu, acupuncture/acupressure; C, control group; E, experimental group; GVM, Governor Vessel moxibustion; RT, rehabilitation training.

### 4.3 Risk of Bias in Included Studies

**FIGURE 3:**
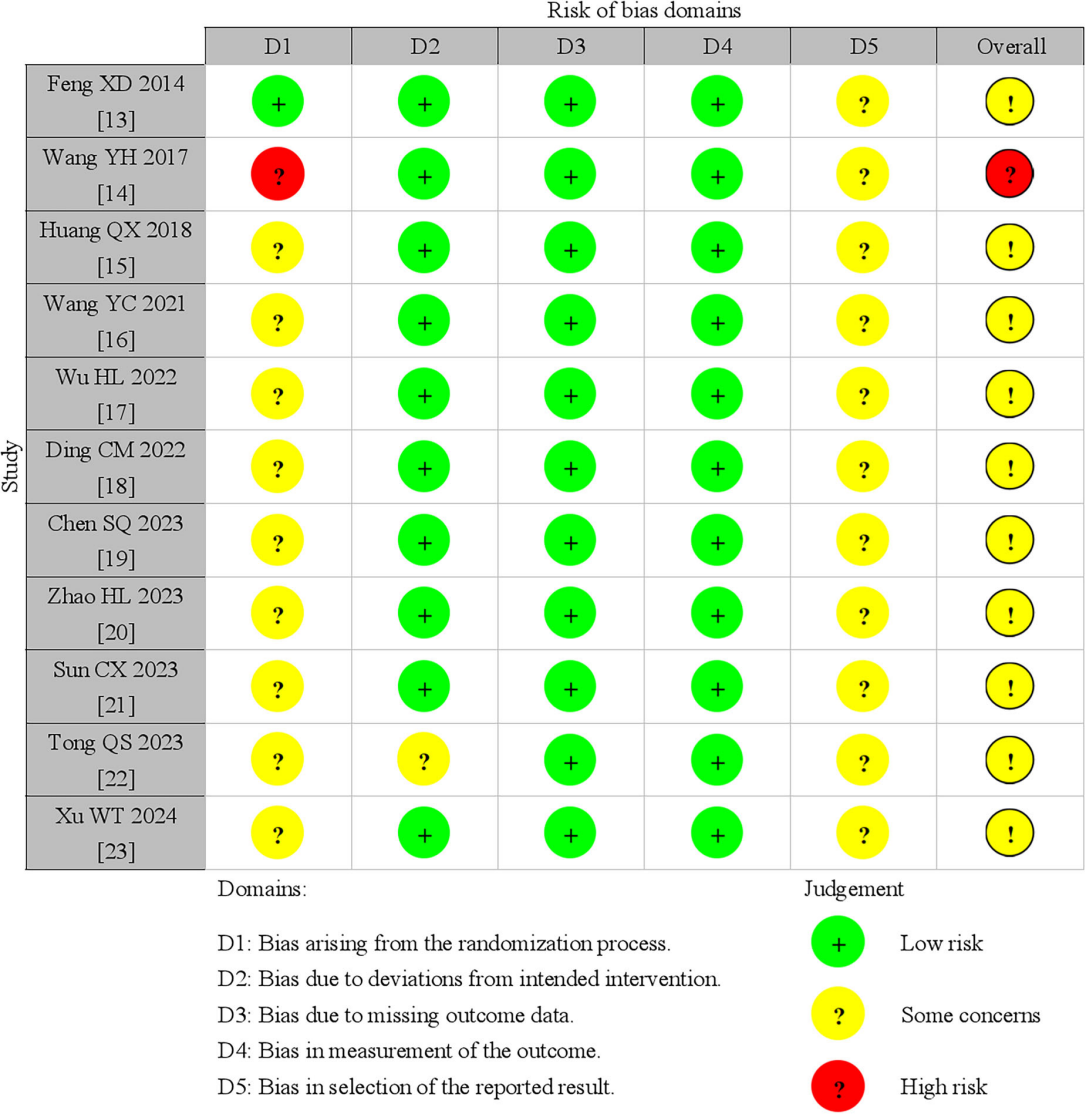
Summary of risk of bias judgments for each study.

A total of 11 RCTs were evaluated using the RoB 2.0 tool. Among them, only one study was identified as having a “high risk” (Wang et al. 2017), and the remaining studies were identified as having “some concerns” (Feng et al. 2014; Huang and Zhong 2018; Wang and Sun 2021; Wu et al. 2022; Ding et al. 2022; Chen et al. 2023; Zhao et al. 2023; Sun and Dong 2023; Tong et al. 2023; Xu and Qin 2024) (Figure 3). In general, the quality of most studies was found to be moderate. Due to insufficient details in the studies, the risk of bias related to allocation concealment remained unclear in nine studies (Huang and Zhong 2018; Wang and Sun 2021; Wu et al. 2022; Ding et al. 2022; Chen et al. 2023; Zhao et al. 2023; Sun and Dong 2023; Tong et al. 2023; Xu and Qin 2024). Specifically, eight studies failed to clarify whether the allocation sequence was concealed (Huang and Zhong 2018; Wang and Sun 2021; Wu et al. 2022; Ding et al. 2022; Chen et al. 2023; Zhao et al. 2023; Sun and Dong 2023; Xu and Qin 2024), and one study did not specify if the allocation was randomized (Tong et al. 2023). One study had a high risk of bias regarding assignment concealment due to its use of simple randomization (Wang et al. 2017). Although none of the studies (Feng et al. 2014; Wang et al. 2017; Huang and Zhong 2018; Wang and Sun 2021; Wu et al. 2022; Ding et al. 2022; Chen et al. 2023; Zhao et al. 2023; Sun and Dong 2023; Tong et al. 2023; Xu and Qin 2024) stated whether participants were aware of their group assignments during the trial, no study proposed additional interventions that deviated from the trial design. Only one RCT reported a dropout from the control group, with a dropout rate significantly below 5% (Tong et al. 2023). All other studies had no dropouts, indicating a low risk of loss‐to‐attrition bias (Feng et al. 2014; Wang et al. 2017; Huang and Zhong 2018; Wang and Sun 2021; Wu et al. 2022; Ding et al. 2022; Chen et al. 2023; Zhao et al. 2023; Sun and Dong 2023; Xu and Qin 2024). Blinding presented significant challenges. Given the nature of moxibustion manipulation, blinding is often impractical. The RoB 2.0 results indicated that descriptions of blinding of participants and personnel were either unclear or low quality. Furthermore, only one RCT noted that assessors were blinded to participant interventions (Tong et al. 2023). In addition, Supporting Information was lacking in all enrolled studies. As a result, it is uncertain whether an analysis plan was established before the start of each RCT (Feng et al. 2014; Wang et al. 2017; Huang and Zhong 2018; Wang and Sun 2021; Wu et al. 2022; Ding et al. 2022; Chen et al. 2023; Zhao et al. 2023; Sun and Dong 2023; Tong et al. 2023; Xu and Qin 2024).

### 4.4 Primary Outcomes

The MAS ranges from 0 to 4, where a higher score indicates a more severe degree of muscle spasticity. And the CSI combines the assessment of tendon jerks, MAS (with the MAS component double‐weighted), and clonus, such that a higher CSI score corresponds to a more pronounced level of spasticity. Both the MAS and CSI have demonstrated relatively good intra‐ and inter‐rater reliability, ensuring a certain level of consistency in the assessment of spasticity among different measurements and between different raters (Vidmar et al. 2023; Gal et al. 2025).

#### 4.4.1 Effect on the MAS

**FIGURE 4:**
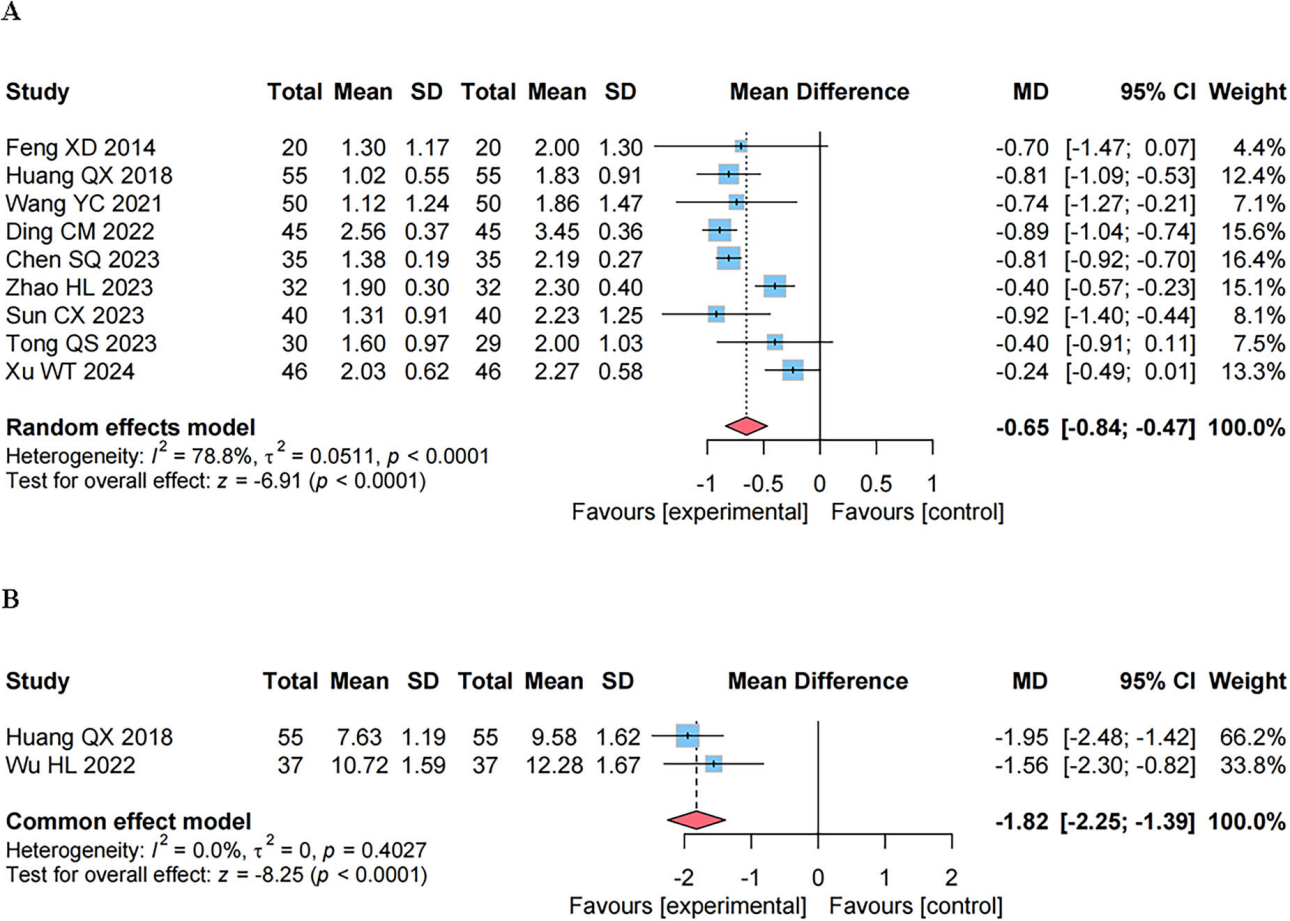
The forest plot of effect on primary outcomes. (A) The modified Ashworth scale (MAS) score. (B) The composite spasticity index (CSI) score.

As shown in Figure 4A, a meta‐analysis of nine studies (Feng et al. 2014; Huang and Zhong 2018; Wang and Sun 2021; Ding et al. 2022; Chen et al. 2023; Zhao et al. 2023; Sun and Dong 2023; Tong et al. 2023; Xu and Qin 2024) involving 705 participants, which used MAS to examine the degree of spasticity, was carried out using a random‐effects model. The meta‐analysis results revealed that, in comparison with the control group (RT administered alone), GVM combined with RT was more effective in decreasing the MAS score (MD, −0.65 [95% CI, −0.84 to −0.47], *p* < 0.01).

##### 4.4.1.1 Sensitivity Analysis

Sensitivity analyses recombined the data by excluding each of the included studies one at a time. The outcome measure included nine studies. As shown in Figure S6, after excluding any single study, the pooled results of the remaining eight studies remained statistically significant. The initial pooled results were minimally influenced by the exclusion of individual studies. This sensitivity analysis, to some extent, suggests that the overall results are robust and stable.

##### 4.4.1.2 Subgroup Analysis

In light of the considerable heterogeneity (*I^2^* = 78.8%), we conducted a more in‐depth subgroup analysis based on several underlying factors that could potentially influence the outcomes derived from real‐world TCM clinical settings. These factors included intervention protocols, treatment time, frequency, area, and duration.

Regarding intervention protocols (*n* = 9), the subgroup analysis showed that the combination of GVM + RT + acupuncture/acupressure (MD, −0.67 [95% CI, −0.96 to −0.39], *p* < 0.00001) was superior to GVM + RT (MD, −0.63 [95% CI, −0.89 to −0.38], *p* < 0.00001) in relieving limb spasticity. Preliminary results suggest that integrating acupuncture/acupressure into the GVM + RT regimen can yield a better synergistic effect (see each subtotal in Figure S1).

In terms of treatment time (*n* = 8), the results showed that, with the assistance of GVM, the MAS score of the experimental group was significantly lower than that of the control group (MD, −0.63 [95% CI, −0.83 to −0.43], *p* < 0.00001). Meanwhile, the heterogeneity within the subgroup was significantly reduced (*I^2^* < 50%). Therefore, the treatment time might be the source of high heterogeneity, as reflected by the heterogeneity of each subgroup presented in Figure S2. Of note, when the treatment time was 60 min, the effect size was the largest (MD, −0.88 [95% CI, −1.03 to −0.73], *p* < 0.00001).

Concerning treatment frequency (*n* = 8), the subgroup analysis showed that the MAS score in the experimental group was significantly lower than that in the control group (MD, −0.63 [95% CI, −0.84 to −0.42], *p* < 0.00001). The heterogeneity was obviously decreased (*I^2^* < 50%), suggesting that the treatment frequency might also be the source of high heterogeneity (see heterogeneity of each subgroup in Figure S3). And the effect size was the largest when the treatment was administered once‐weekly (MD, −0.87 [95% CI, −1.02 to −0.73], *p* < 0.00001).

In terms of treatment area (*n* = 9), the subgroup analysis showed that the effect size was greater in the upper limb (MD, −0.83 [95% CI, −0.91 to −0.74], *p* < 0.00001) compared to the lower limb (MD, −0.24 [95% CI, −0.49 to 0.01], *p* = 0.06) and the combined upper and lower limbs (MD, −0.60 [95% CI, −0.93 to −0.27], *p* = 0.0003), with the subtotal data for each subgroup presented in Figure S4.

Regarding treatment duration (*n* = 9), subgroup analysis revealed that the experimental group outperformed the control group in relieving limb spasticity (MD, −0.65 [95% CI, −0.84 to ‐0.47], *p* < 0.00001; see total in Figure S5). More importantly, the largest effect size was observed when treatment duration was 8 weeks or longer (MD, −0.81 [95% CI, −0.92 to ‐0.70], *p* < 0.00001; see subtotal of the third subgroup in Figure S5). All results of the subgroup analyses are summarized in Figures S1–S5.

#### 4.4.2 Effect on the CSI

The CSI analysis included two studies involving a total of 184 participants (Huang and Zhong 2018; Wu et al. 2022), with one high‐risk study excluded (Wang et al. 2017). A fixed‐effects model was adopted for this analysis. Results indicated that, compared with RT used alone, the combination of GVM and RT exhibited significant potential in alleviating lower limb spasticity, as evidenced by a reduced CSI score (MD, −1.82 [95% CI, −2.25 to −1.39], *p* < 0.01) (Figure 4B).

### 4.5 Secondary Outcomes

#### 4.5.1 Effect on the ER

**FIGURE 5:**
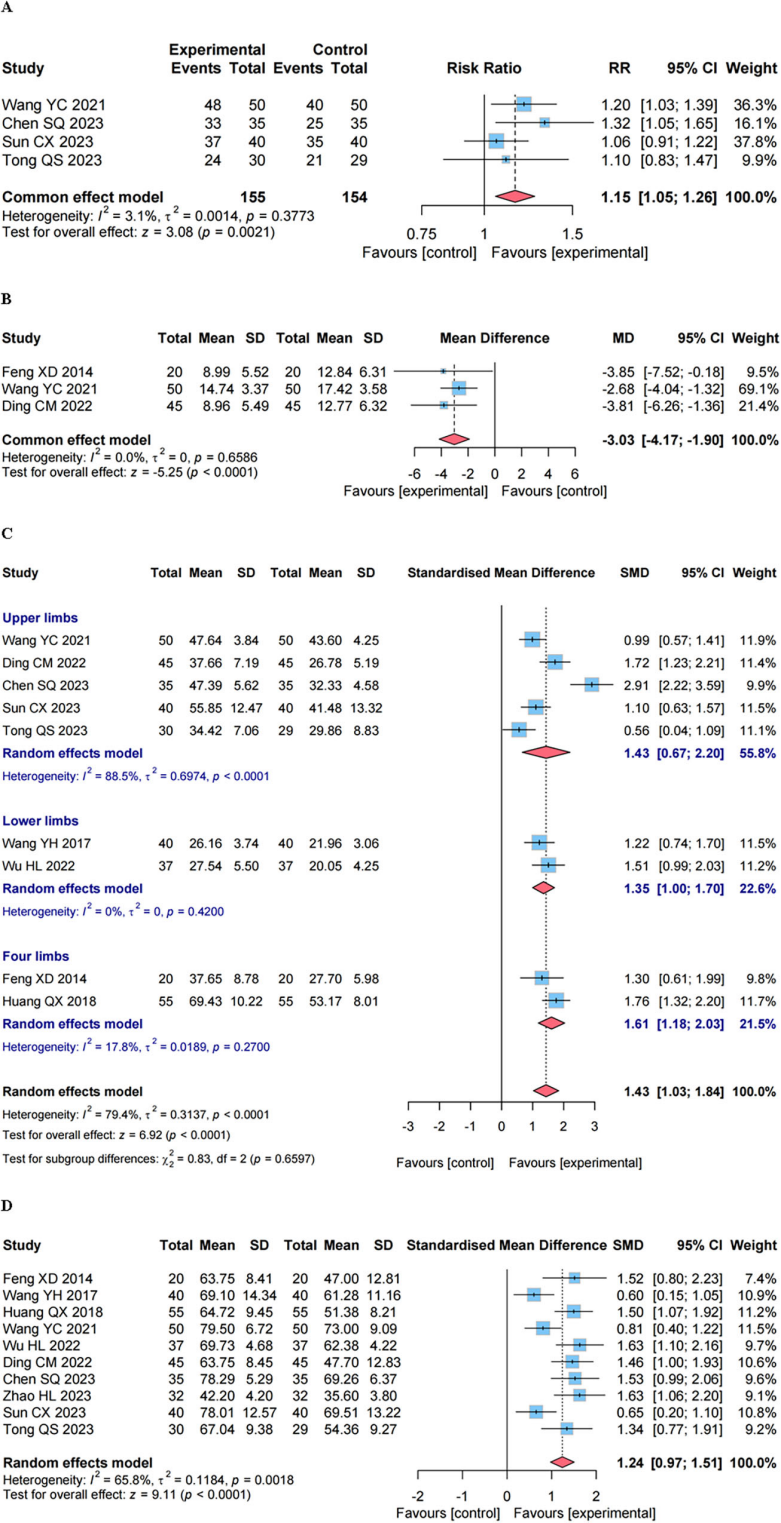
The forest plot of effect on secondary outcomes. (A) The effective rate (ER). (B) The root mean square (RMS) value of surface electromyography. (C) The Fugl–Myer assessment (FMA) score. (D) The Barthel Index (BI)/modified Barthel Index (MBI) score.

As presented in Figure 5A, four studies (Wang and Sun 2021; Chen et al. 2023; Sun and Dong 2023; Tong et al. 2023) utilized fixed‐effects model to calculate the ER of PSS in 309 patients. The results revealed that, in contrast to the control group, GVM combined with RT could obviously improve the ER (RR, 1.15 [95% CI, 1.05 to1.26], *p* < 0.01).

#### 4.5.2 Effect on the RMS Value

As depicted in Figure 5B, three studies (Feng et al. 2014; Wang and Sun 2021; Ding et al. 2022) employed the RMS value of surface electromyography (sEMG) to assess the biceps brachii muscle tone in 230 patients with PSS. Given the low heterogeneity among the studies (*I^2^* = 0%), a fixed‐effects model was adopted. The results demonstrated that the combination of GVM and RT could effectively reduce RMS value of the biceps brachii on the affected side of PSS patients (MD, −3.03 [95% CI, −4.17 to −1.90], *p* < 0.01), which further proved that this combined therapy wielded preferable antispastic effects.

#### 4.5.3 Effect on the FMA

As presented in Figure 5C, nine studies (Feng et al. 2014; Wang et al. 2017; Huang and Zhong 2018; Wang and Sun 2021; Wu et al. 2022; Ding et al. 2022; Chen et al. 2023; Sun and Dong 2023; Tong et al. 2023) utilized the FMA to evaluate the motor functions of patients with PSS, where a higher FMA score indicates better motor function. Initially, without site‐specific categorization, there was a high degree of heterogeneity (*I^2^* = 79.4%). Subsequent subgroup analysis by assessment sites (upper limbs, lower limbs, and four limbs) revealed that although high heterogeneity persisted in some subgroups (e.g., upper limbs, *I^2^* = 88.5%) as well as in the overall reclassified analysis, the results consistently showed that the experimental group had a more significant improvement in FMA scores compared to the control group (SMD, 1.43 [95% CI, 1.03–1.84], *p* < 0.01). This indicates that regardless of whether the analysis was conducted overall or stratified by assessment sites, despite high heterogeneity, the experimental group's superiority over the control group in motor function improvement was significant. Of note, when two studies with a substantial impact on heterogeneity were excluded (Ding et al. 2022; Chen et al. 2023), the heterogeneity of the upper limbs decreased significantly (*I^2^* from 88.5% to 19%; see Figure S11). This initial high heterogeneity may be attributed to two factors: the relatively small number of included studies, which amplifies the influence of individual study variations and the inclusion of three gray literature studies.

#### 4.5.4 Effect on the BI/MBI

As shown in Figure 5D, 10 studies (Feng et al. 2014; Wang et al. 2017; Huang and Zhong 2018; Wang and Sun 2021; Wu et al. 2022; Ding et al. 2022; Chen et al. 2023; Zhao et al. 2023; Sun and Dong 2023; Tong et al. 2023) incorporated the BI/MBI. Of note, a higher BI/MBI score indicates better functional ability in daily life. Despite the presence of significant heterogeneity among these studies (*I^2^* = 65.8%), the results indicated that a more substantial increase in BI/MBI scores was found in the experimental group when compared to the control group (SMD, 1.24 [95% CI, 0.97–1.51], *p* < 0.01). This, in turn, implies that the combination of GVM and RT achieved more favorable improvements in the daily living activity of patients with PSS.

#### 4.5.5 Effect on the AE

Only one study (Chen et al. 2023) reported a GVM‐related AE in the treatment group (*n* = 430), with an incidence of 1/430. Specifically, one patient in the treatment group developed local skin reactions (subcutaneous redness and itching) at the moxibustion site—classified as mild, as it did not impair daily activities or require additional medical evaluation. This event was deemed directly related to GVM, given its occurrence at the intervention site and the absence of confounding factors (e.g., preexisting skin lesions and skin‐irritating medications). No special medical intervention was administered; symptoms resolved spontaneously and completely without residual discomfort, and did not affect the overall study conduct and results.

#### 4.5.6 Sensitivity Analysis on Secondary Outcomes

As shown in Figures S7–S10, the sensitivity analyses for ER, RMS value, FMA, and BI/MBI demonstrated that the results appeared to be robust.

### 4.6 Publication Bias

**FIGURE 6:**
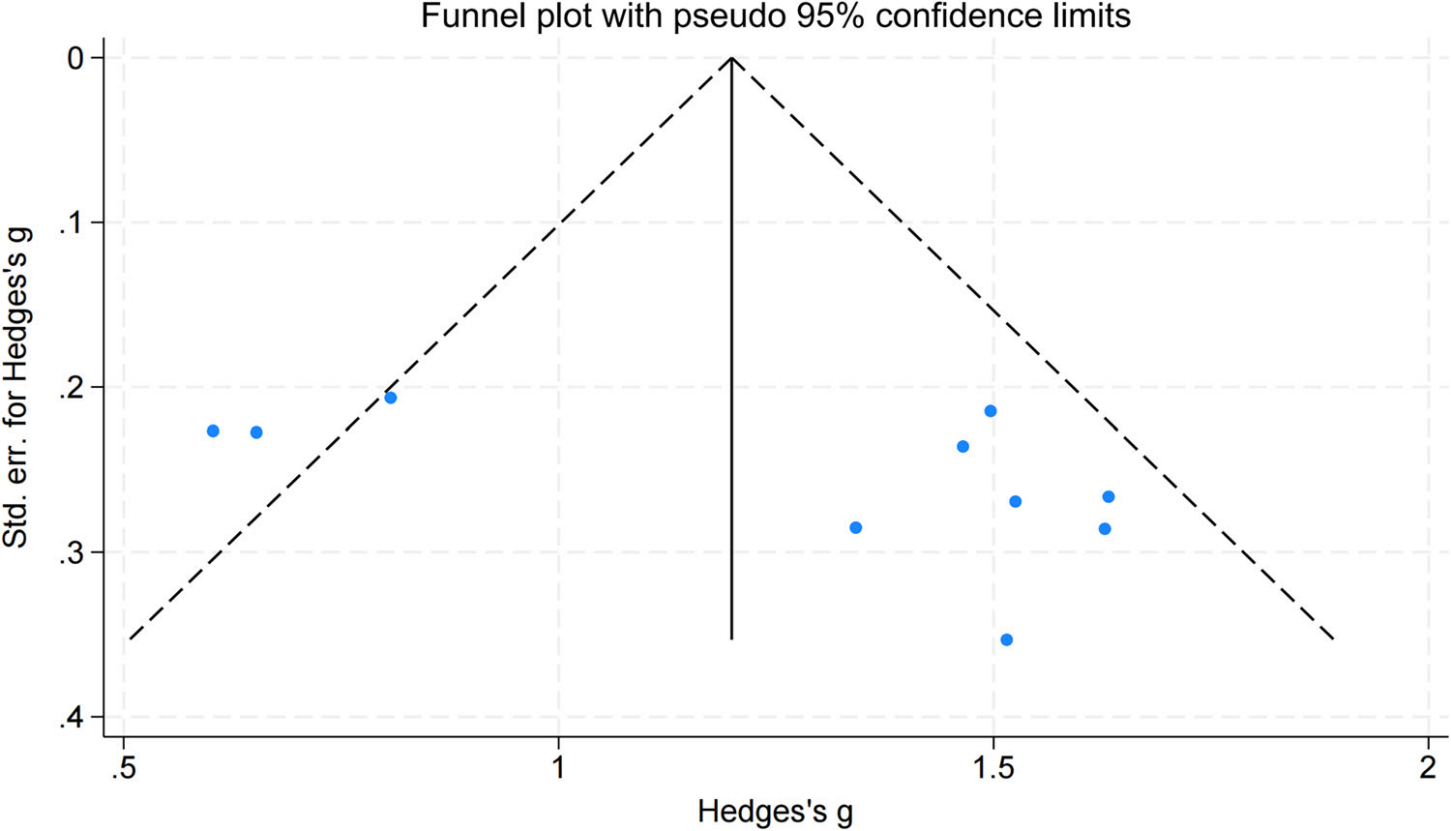
The funnel plot of publication bias of BI/MBI.

To assess publication bias for the BI/MBI—reported in 10 studies (Feng et al. 2014; Wang et al. 2017; Huang and Zhong 2018; Wang and Sun 2021; Wu et al. 2022; Ding et al. 2022; Chen et al. 2023; Zhao et al. 2023; Sun and Dong 2023; Tong et al. 2023)—we performed Egger's (*p* = 0.0686) and Begg's tests (*p* = 0.3711), both showing no significant bias (Figure 6). The funnel plot revealed most data points clustered within pseudo‐95% confidence limits, suggesting general symmetry. However, one side appeared sparse—likely due to low power in the small sample (*n* = 10) or true effect variability. Our search included eight major databases for comprehensiveness, yet three grey literature references in Zhao et al. (2023) remained unretrievable, introducing uncertainty. Despite statistical support, we interpret results cautiously; the small sample and unretrievable grey literature may reduce asymmetry detection power and hide potential bias.

### 4.7 Evidence Evaluation

The certainty of the evidence was critically evaluated. The results indicated that the evidence for “ER” was rated as moderate quality, which was ascribed to the existence of a risk of bias in the relevant studies. For five outcomes, the evidence was rated as low‐quality. Specifically, “MAS,” “BI/MBI,” and “FMA” were given this rating due to both risks of bias and inconsistency across the studies. Meanwhile, the “RMS” value was deemed low‐quality evidence due to risks of bias and imprecision in data. “CSI” was considered to be very low‐quality evidence. A comprehensive summary of the GRADE classification results is summarized in Table 2.

**TABLE 2:** GRADE classification for evidence evaluation.

Outcomes	No. of studies	Certainty assessment						No. of patients		Effect		Certainty	Importance
		Study design	Risk of bias	Inconsistency	Indirectness	Imprecision	Other considerations	E	C	Relative (95% CI)	Absolute (95% CI)		
MAS	9	RCTs	Serious^a^	Serious^b^	Not serious	Not serious	None	353	352	—	MD 0.65 lower (0.84 lower to 0.47 lower)	⨁⨁◯◯ Low^a,b^	Critical
CSI	3	RCTs	Serious^a^	Very serious^b^	Not serious	Serious^c^	None	132	132	—	MD 1.51 lower (2.09 lower to 0.93 lower)	⨁◯◯◯ Very low^a,b,c^	Critical
ER	4	RCTs	Serious^a^	Not serious	Not serious	Not serious	None	142/155 (91.6%)	121/154 (78.6%)	RR 1.15 (1.05–1.27)	118 more per 1000 (from 39 more to 212 more)	⨁⨁⨁◯ Moderate^a^	—
RMS	3	RCTs	Serious^a^	Not serious	Not serious	Serious^b^	None	115	115	—	MD 3.03 lower (4.17 lower to 1.9 lower)	⨁⨁◯◯ Low^a,b^	—
FMA	9	RCTs	Serious^a^	Serious^b^	Not serious	Not serious	None	352	351	—	SMD 1.43 higher (1.05 higher to 1.8 higher)	⨁⨁◯◯ Low^a,b^	—
BI/MBI	10	RCTs	Serious^a^	Serious^b^	Not serious	Not serious	None	384	383	—	SMD 1.24 SD higher (0.97 higher to 1.51 higher)	⨁⨁◯◯ Low^a,b^	—

Abbreviations: BI/MBI, Barthel Index/modified Barthel Index; C, control group; CI, confidence interval; CSI, composite spasticity index; E, experimental group; ER, effective rate; FMA, Fugl–Myer assessment; MAS, modified Ashworth scale; MD, mean difference; RCTs, randomized controlled trials; RMS, root mean square value; RR, risk ratio; SMD, standardized mean difference.

^a^Lack of blinding.

^b^ *I^2^* value of the combined results was large and high heterogeneity.

^c^Small sample size and the confidence intervals were wide.

## 5 Discussion

### 5.1 Summary of Key Findings

This systematic review and meta‐analysis of 11 RCTs involving 859 PSS patients is, to our knowledge, the first to rigorously evaluate the efficacy of GVM combined with routine RT for PSS. The results consistently demonstrate that GVM, as a distinct complementary therapy to RT, significantly alleviates muscle spasticity—evidenced by reduced scores on the MAS (MD, −0.65 [95% CI, −0.84 to −0.47], *p* < 0.01) and CSI (MD, −1.82 [95% CI, −2.25 to −1.39], *p* < 0.01)—and improves objective indicators such as the RMS value of sEMG. Additionally, GVM plus RT enhances motor function (higher FMA scores) and activities of daily living (higher BI/modified BI scores). AEs were minimal (only one case of transient subcutaneous redness), indicating GVM's favorable safety profile.

Subgroup analyses further revealed that treatment parameters (60‐min sessions, once‐weekly frequency, and ≥ 8‐week duration) optimized GVM's efficacy. Additionally, the results revealed that GVM exerted a significant effect on upper‐limb spasticity, while its effect on lower‐limb spasticity was not statistically significant. These findings collectively support the clinical value of GVM as an adjunct to RT for PSS.

### 5.2 Characteristics of GVM and Its Association With GV

GVM is an important technique in TCM with unique characteristics that distinguish it from conventional moxibustion. Administered primarily via ginger‐partitioned moxibustion along the spinal segments of GV, GVM combines three key elements: physical thermal stimulation from burning moxa, pharmacological properties of moxa wool (volatile oils with anti‐inflammatory effects), and synergistic action of ginger (rich in gingerol, which enhances analgesic and anti‐inflammatory effects) (Zhang et al. 2017; Lu et al. 2022). These attributes endow GVM with a large treatment area, thick moxa cones, and a potent thermal effect.

In TCM theory, the GV is revered as the “sea of yang meridians,” governing the integration of cerebral and spinal functions and regulating the balance of yin–yang and qi–blood circulation (Liao et al. 2023). Pathologically, PSS is attributed to qi stagnation, blood stasis, and cold pathogenic factors invading the meridians–sinews, leading to muscle spasticity (Wang et al. 2019). GVM's thermal stimulation along the GV directly targets these imbalances; it warms the meridians to dispel cold, promotes qi and blood circulation, and soothes tendons to relieve spasticity—aligning with TCM principles of treating brain disorders through GV regulation (Zhang et al. 2017; Liao et al. 2023).

### 5.3 Potential Antispastic Mechanisms of Stimulation on the Spinal Segments of GV

While the precise mechanisms underlying spasticity alleviation via stimulation of the GV spinal segments remain to be fully elucidated, existing evidence identifies multiple pathways mediating this therapeutic effect. Neural regulation: Stimulation of GV‐acupoints activates motor‐related brain regions (e.g., precentral gyrus and supplementary motor area) (He 2014) and restores the balance between excitatory (glutamate) and inhibitory (GABA) neurotransmitters, a process critical for reducing abnormal neural excitability in PSS (Li et al. 2022; Wang et al. 2023). Muscle function improvement: By stimulating the GV spinal segments, GVM reduces the RMS values of sEMG in the spastic biceps brachii (Wang and Sun 2021), a direct indicator of reduced muscle hypertonia. Anti‐inflammatory effects: Moxa combustion products and gingerol inhibit the production of inflammatory mediators in poststroke brain tissue (Zhang et al. 2017; Zhou et al. 2022), thereby alleviating secondary brain damage and facilitating neural recovery. Collectively, these interconnected mechanisms partly explain why GVM exerts effective antispastic effects in PSS patients.

### 5.4 Limitations and Implications for Future Research

Despite promising findings, this study has several limitations. First, all included studies were conducted within China, which may introduce certain regional constraints. Second, the sample size of the included studies is relatively limited, which may further impose constraints on the generalizability of our findings to patient groups with greater heterogeneity. Third, high heterogeneity across studies hinders direct comparisons—this stems from variations in treatment protocols (e.g., session time, frequency, and total duration), differences in participant characteristics, and cumulative disparities in study designs across the included studies. Fourth, GVM's physical nature makes participant and personnel blinding challenging, introducing potential performance bias; additionally, most studies lack long‐term follow‐up, which limits our ability to draw conclusions about the sustained efficacy of GVM. Finally, although scales such as the MAS and CSI are clinically reliable, their subjective scoring nature may introduce evaluator bias.

To address these gaps and facilitate clinical translation, the following recommendations for future research are proposed. First, develop consensus guidelines for optimal GVM parameters in PSS management to reduce heterogeneity. Second, integrate objective assessment techniques into research frameworks; functional magnetic resonance imaging (fMRI) can evaluate GVM's effects on brain neurological regulation (Mayorova et al. 2023), sEMG enables precise measurement of muscle electrical activity during spasticity, and musculoskeletal ultrasound can detect changes in muscle properties (Wang and Sun 2021; Chen et al. 2021). Third, conduct multicenter RCTs with long‐term follow‐up, include diverse populations, and incorporate patient‐reported outcomes (e.g., pain and quality of life) for holistic assessment (Ramesh et al. 2023).

Given that RT—a cornerstone of PSS management that promotes neuroplasticity and motor function via task‐oriented exercises—synergizes with GVM to yield superior outcomes than RT alone, a flexible, personalized rehabilitation protocol is recommended (Figure 1C). Early rehabilitation (initiated safely 24–72 h poststroke and sustained for at least 3 months) is critical, as human neuroplasticity peaks within 3 months poststroke (Coleman et al. 2017; Wang et al. 2023); physical therapies like compression, weight‐bearing, and stretching can be gradually introduced (Zhang et al. 2024), with TCM practitioners guiding early in‐bed exercises to build tolerance progressively (Huang et al. 2022). In the chronic recovery stage, the focus should shift from passive to active movements; range‐of‐motion training to maintain joint mobility (Pedrosa et al. 2022) and aerobic exercises to enhance cardiovascular function (Hill et al. 2023) are particularly beneficial, as these active interventions promote adaptive neuroplasticity, restore correct movement patterns, and ultimately alleviate spasticity.

## 6 Conclusion

GVM serves as an effective and safe complementary intervention to RT for poststroke muscle spasticity. However, the relatively high risk of bias and heterogeneity across current studies mean GVM's efficacy as a complementary therapy still requires in‐depth verification. Future research should focus on standardization, mechanistic clarification, and long‐term efficacy to establish evidence‐based recommendations.

## Author Contributions

**Jun‐Xiang Wang**: conceptualization, writing – original draft, writing – review and editing, methodology, supervision, funding acquisition. **Zhe‐Hao Hu**: writing – original draft, data curation, methodology, software. **Yi‐Yang Song**: writing – original draft, data curation, methodology, software. **Jin‐Shan Zhong**: methodology, validation. **Miao He**: validation.

## Funding

This research was supported by the National Natural Science Foundation of China (grant no. 82405579).

## Ethics Statement

The authors have nothing to report.

## Conflicts of Interest

The authors declare no conflicts of interest.

## Supporting information




**Supplementary Material**: brb371346‐supp‐0001‐SuppMat.docx

## Data Availability

The data underpinning this study are available in the article and in its Supporting Information.
