# Supplementary material for: New Perspectives on the Efficacy of Governor Vessel Moxibustion Combined With Rehabilitation Training for Poststroke Muscle Spasticity: A Systematic Review and Meta‐Analysis of Randomized Controlled Trials
Source: Brain Behav. 2026 Mar 25;16(4):e71346. doi: 10.1002/brb3.71346 (PMC13111996; doi:10.1002/brb3.71346)
Supplement: Supplementary file 1 — Supplementary Material: brb371346‐supp‐0001‐SuppMat.docx [file BRB3-16-e71346-s001.docx]

**New Perspectives on the Efficacy of Governor Vessel Moxibustion Combined with Rehabilitation Training for Post-Stroke Muscle Spasticity: A Systematic Review and Meta-Analysis of Randomized Controlled Trials**


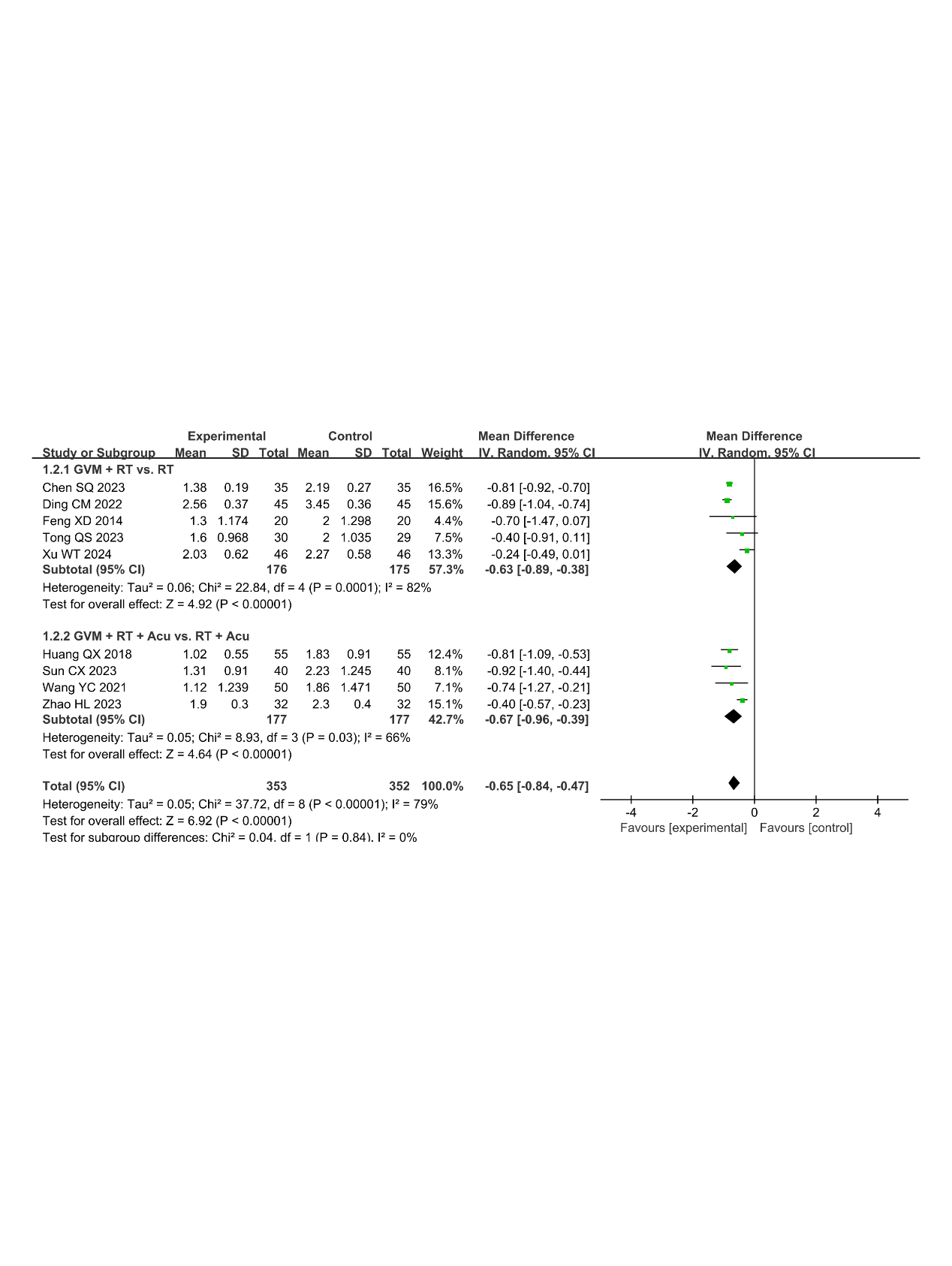


**Supplementary Fig. 1.** The forest plot of subgroup analysis of intervention protocols. Note: GVM, Governor Vessel moxibustion; RT, rehabilitation training; Acu, acupuncture/acupressure.


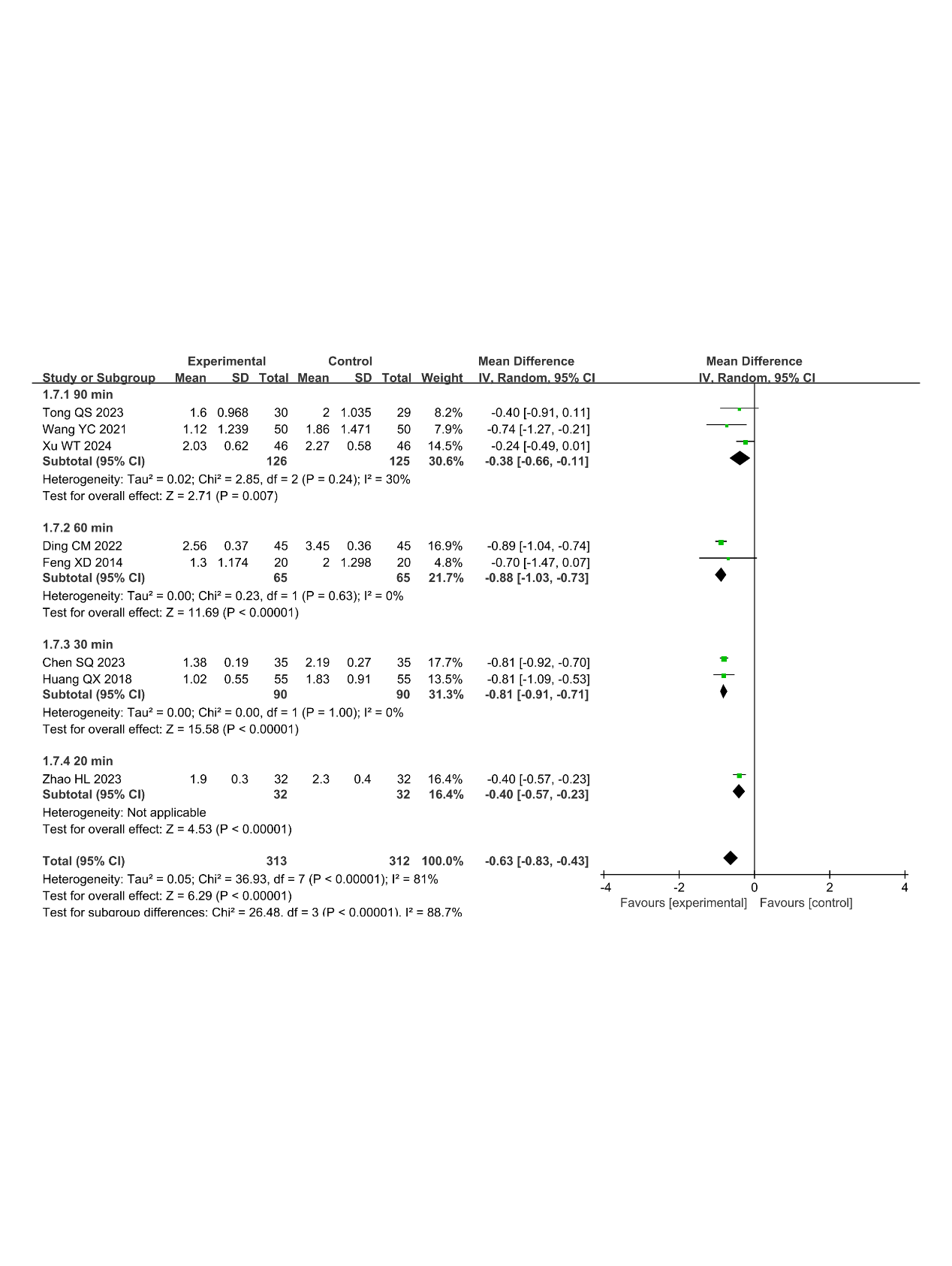


**Supplementary Fig. 2.** The forest plot of subgroup analysis of treatment time.


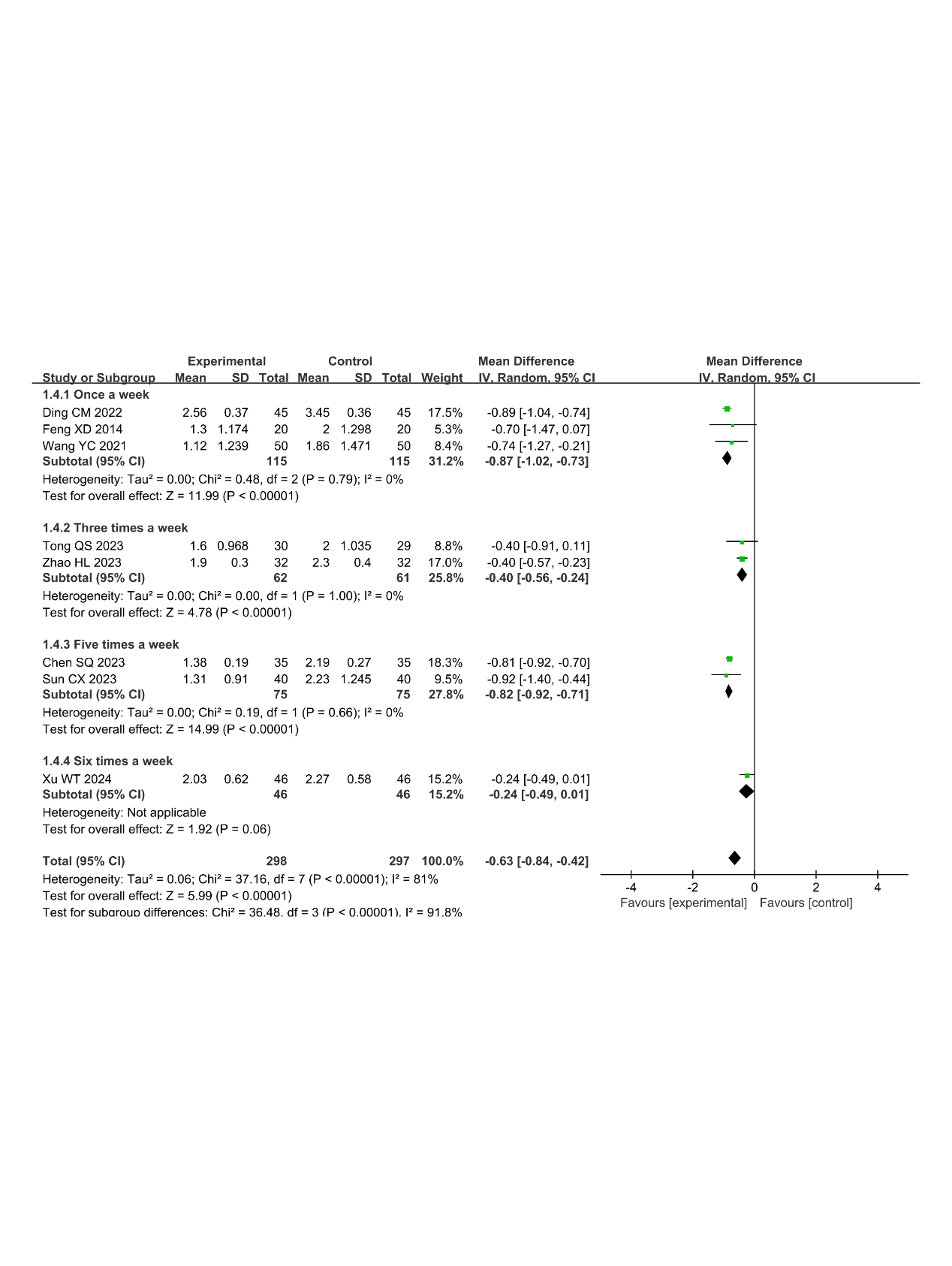


**Supplementary Fig. 3.** The forest plot of subgroup analysis of treatment frequency.


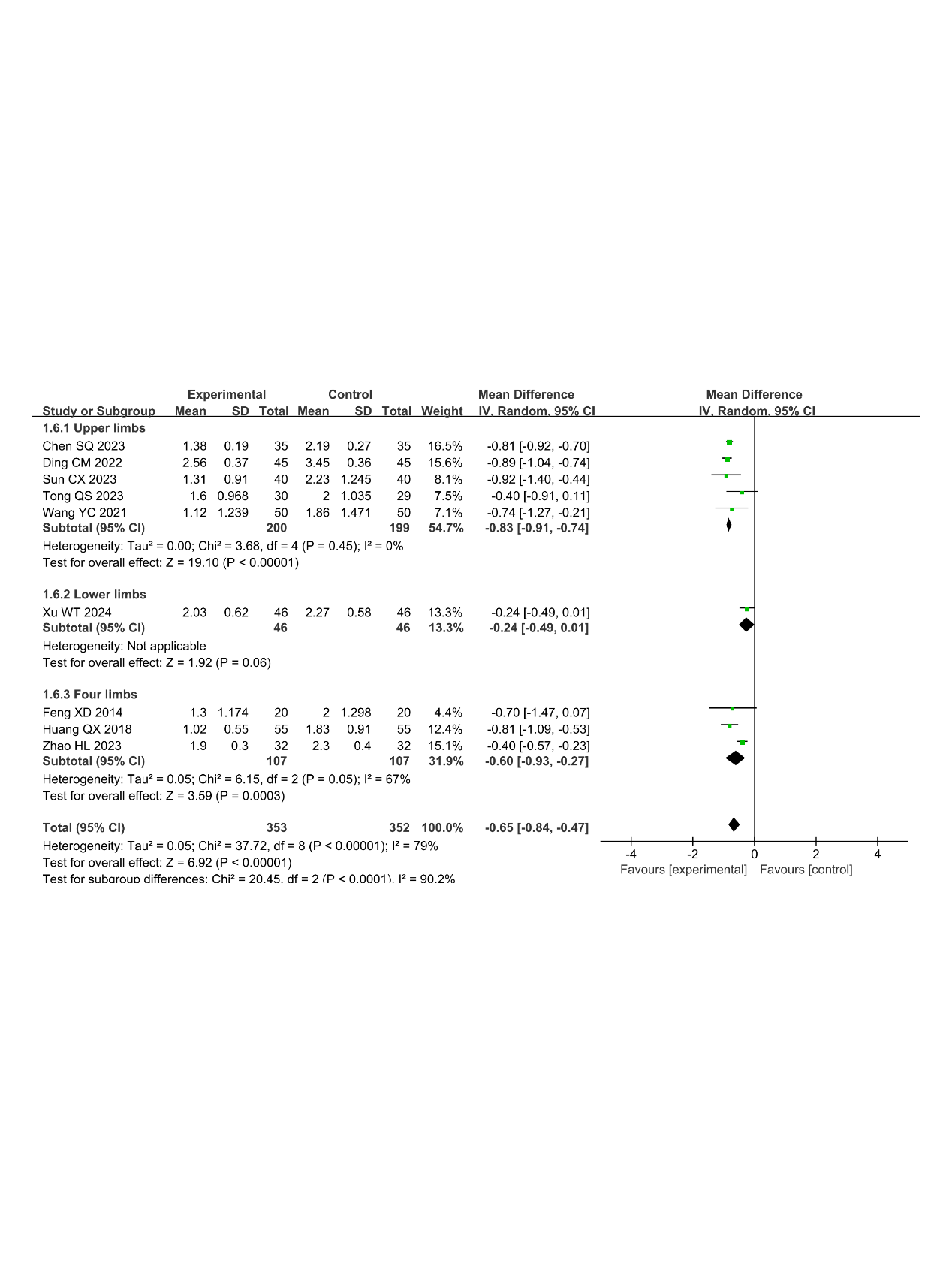


**Supplementary Fig. 4.** The forest plot of subgroup analysis of treatment area.


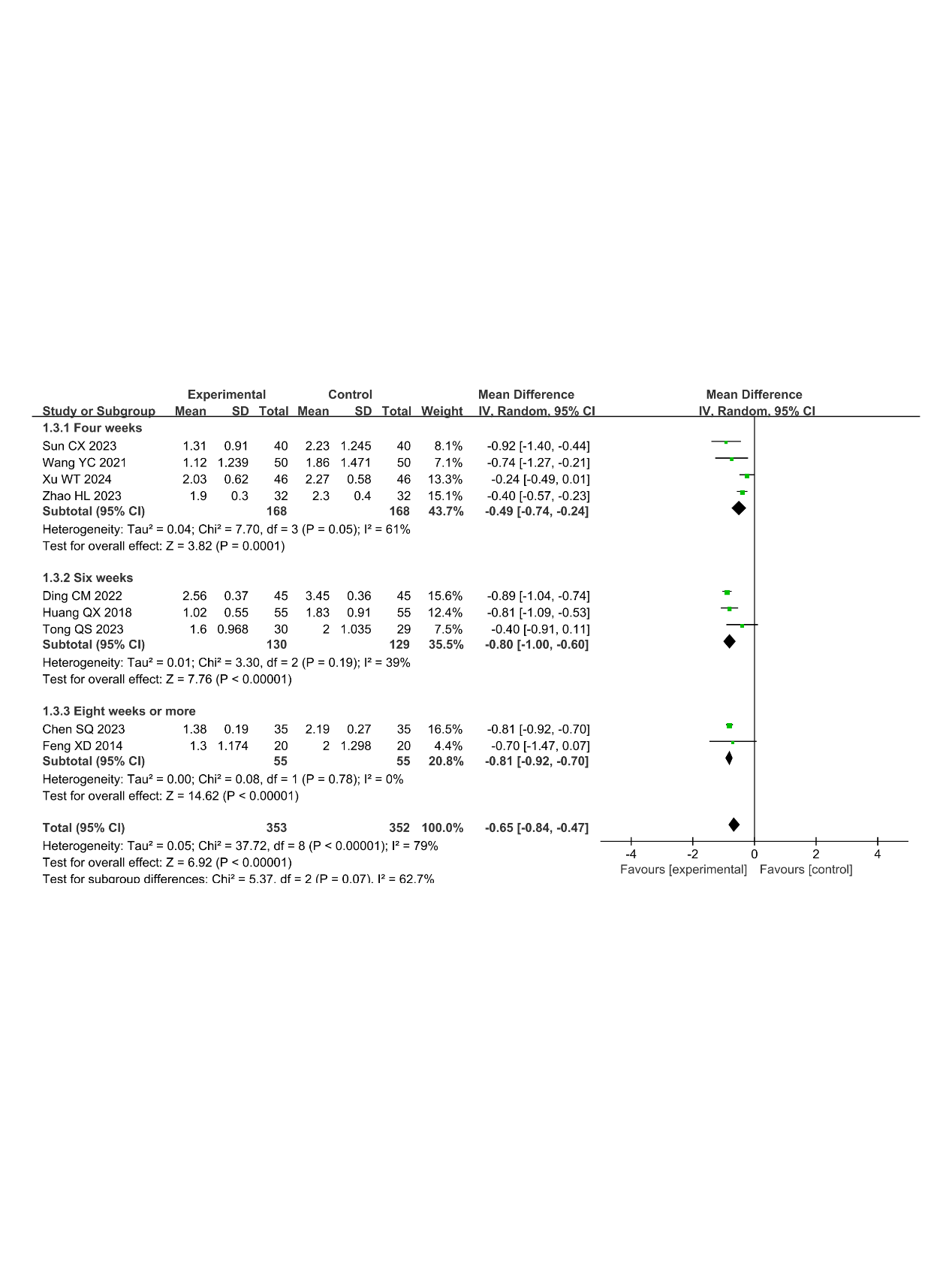


**Supplementary Fig. 5.** The forest plot of subgroup analysis of treatment duration.


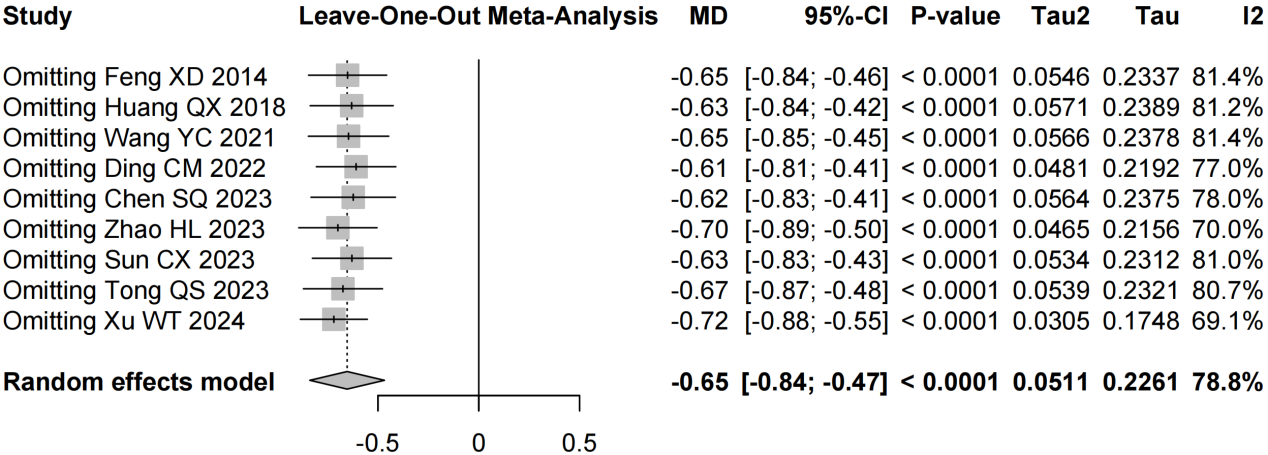


**Supplementary Fig. 6.** The forest plot of sensitivity analysis of modified Ashworth scale (MAS) score.


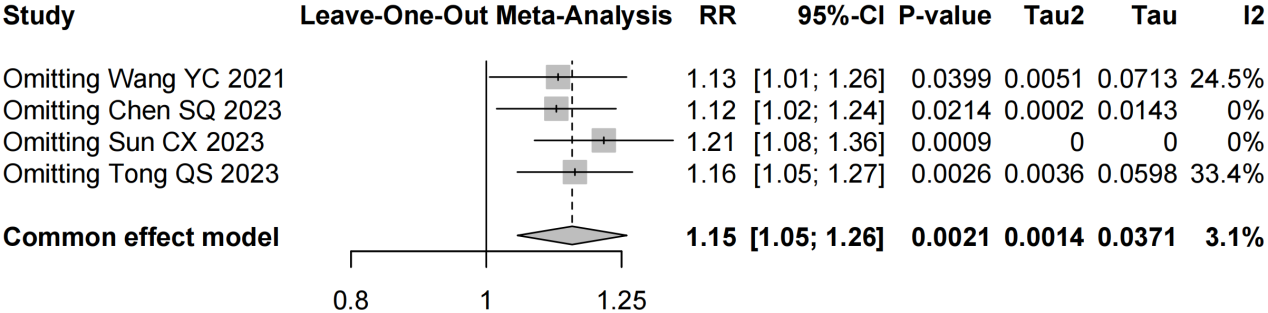


**Supplementary Fig. 7.** The forest plot of sensitivity analysis of effective rate (ER).


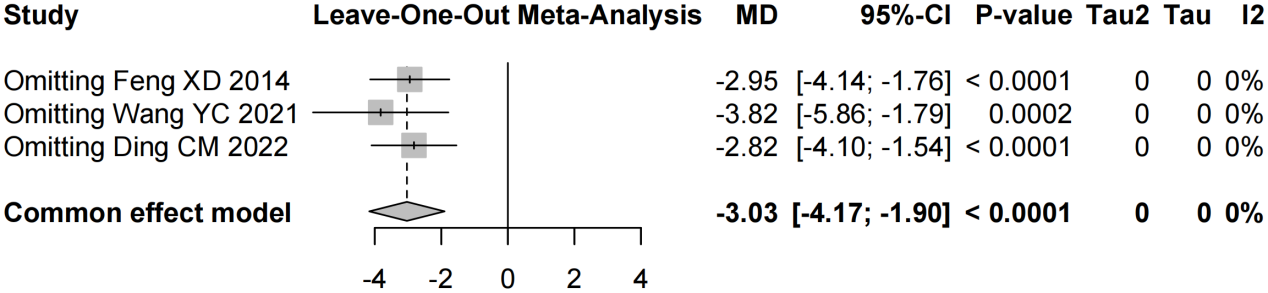


**Supplementary Fig. 8.** The forest plot of sensitivity analysis of root mean square (RMS) value of surface electromyography.


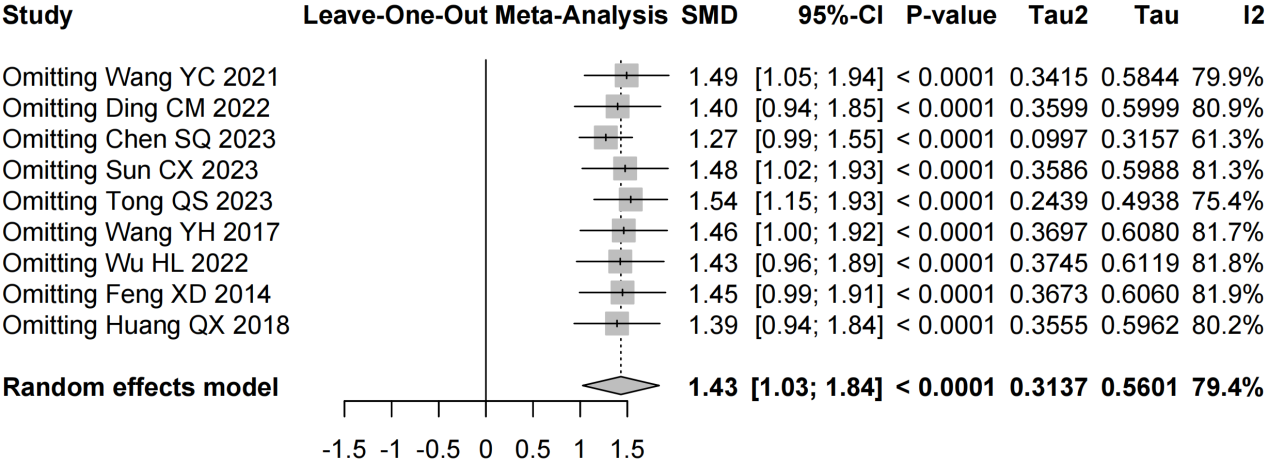


**Supplementary Fig. 9.** The forest plot of sensitivity analysis of Fugl-Myer assessment (FMA) score.


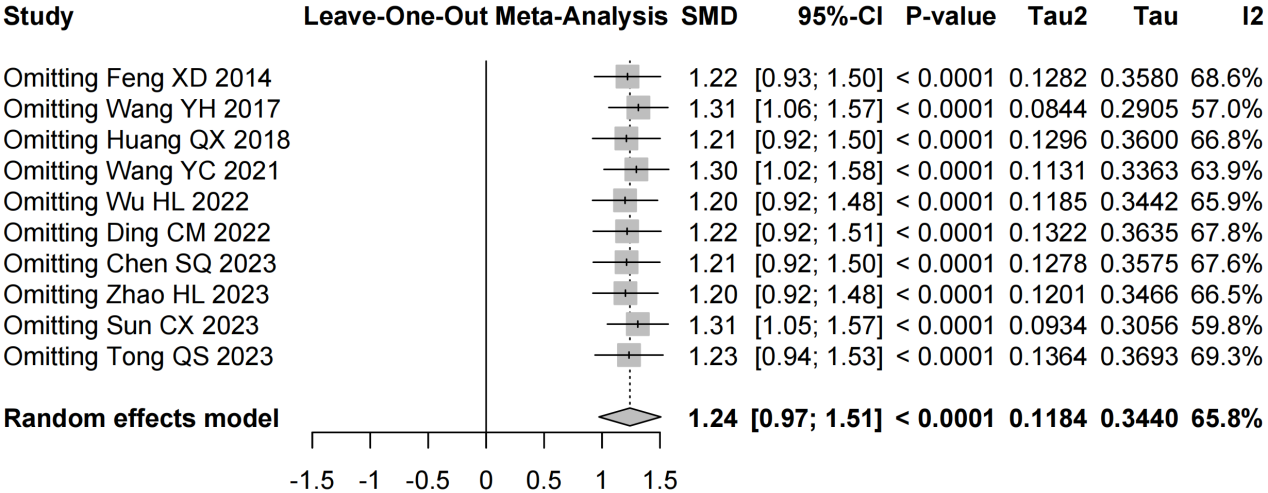


**Supplementary Fig. 10.** The forest plot of sensitivity analysis of Barthel Index (BI)/modified Barthel Index (MBI) score.


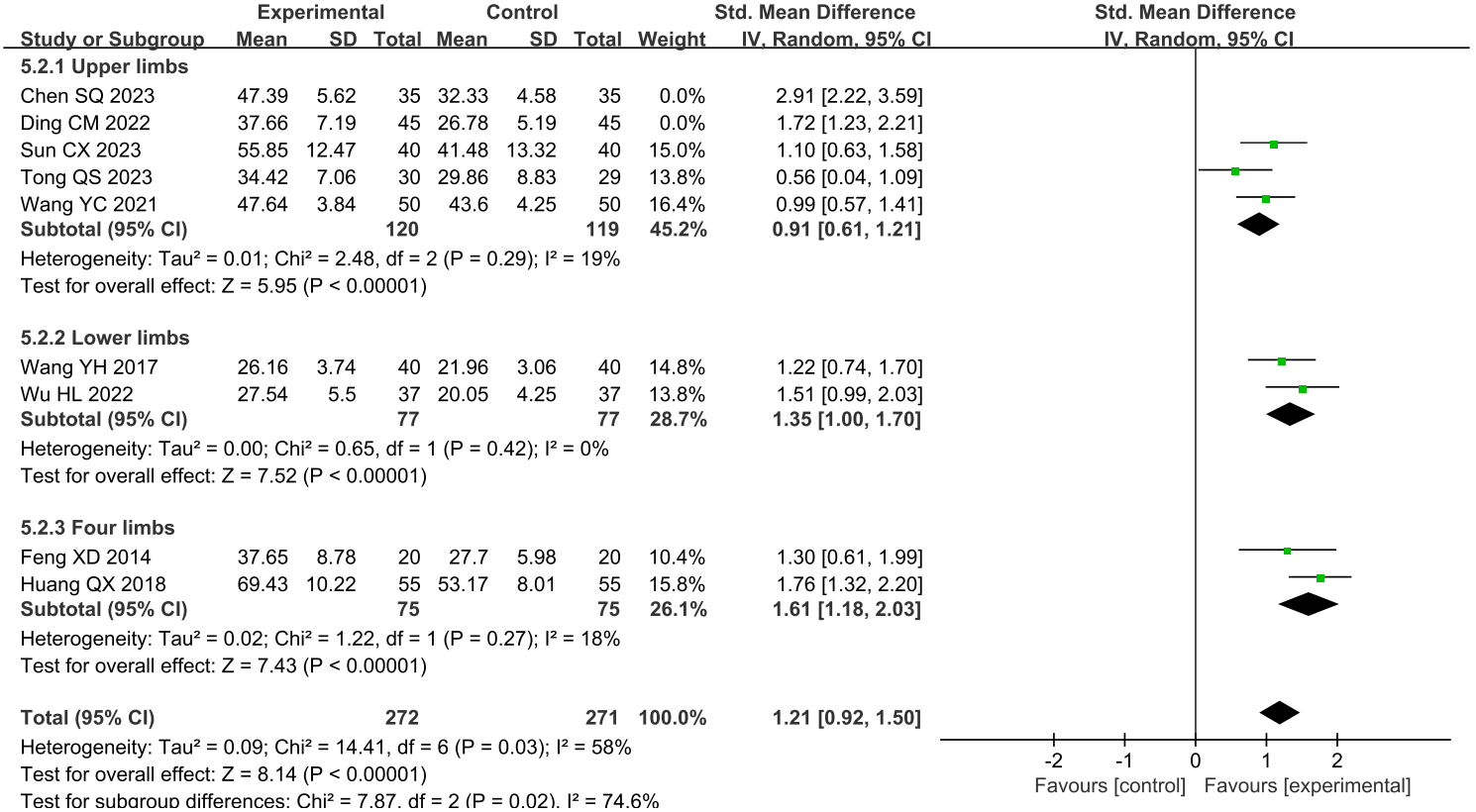


**Supplementary Fig. 11.** The forest plot of effect on FMA after excluding [Chen SQ 2023] and [Ding CM 2022].

**Search Strategies**

*S1*

Cochrane 27

Search Name:

Date Run: 29/09/2024 05:35:23

Comment:

ID Search Hits

#1 MeSH descriptor: [Moxibustion] explode all trees 689

#2 MeSH descriptor: [Stroke Volume] explode all trees 5814

#3 MeSH descriptor: [Cerebral Hemorrhage] explode all trees 1597

#4 MeSH descriptor: [Cerebral Infarction] explode all trees 1685

#5 MeSH descriptor: [Brain Infarction] explode all trees 1905

#6 #2 or #3 or #4 or #5 9213

#7 MeSH descriptor: [Muscle Tonus] explode all trees 364

#8 MeSH descriptor: [Hemiplegia] explode all trees 995

#9 MeSH descriptor: [Paralysis] explode all trees 2897

#10 MeSH descriptor: [Muscle Spasticity] explode all trees 1299

#11 MeSH descriptor: [Spasm] explode all trees 793

#12 MeSH descriptor: [Muscle Cramp] explode all trees 282

#13 MeSH descriptor: [Convulsants] explode all trees 7

#14 #7 or #8 or #9 or #10 or #11 or #12 or #13 5302

#15 #1 #14 27

*S2*

Embase(98)

Session Results

.......................................................

No. Query Results Results Date

#19. #18 AND 'randomized controlled trial'/de 98 28 Sep 2024

#18. #17 AND ('Article'/it OR 'Article in Press'/it) 174 28 Sep 2024

#17. #1 AND #15 AND #16 209 28 Sep 2024

#16. #12 OR #13 OR #14 678,711 28 Sep 2024

#15. #2 OR #3 OR #4 OR #5 OR #6 OR #7 OR #8 OR #9 OR 571,910 28 Sep 2024

#10 OR #11

#14. 'brain infarction'/exp 96,117 28 Sep 2024

#13. 'brain hemorrhage'/exp 197,746 28 Sep 2024

#12. 'cerebrovascular accident'/exp 467,806 28 Sep 2024

#11. 'muscle twitch'/exp 8,202 28 Sep 2024

#10. 'convulsion'/exp 42,006 28 Sep 2024

#9. 'muscle cramp'/exp 23,047 28 Sep 2024

#8. 'muscle spasm'/exp 97,850 28 Sep 2024

#7. 'paralysis'/exp 413,285 28 Sep 2024

#6. 'hemiplegia'/exp 25,066 28 Sep 2024

#5. 'muscle tightness'/exp 798 28 Sep 2024

#4. 'hemiparesis'/exp 29,614 28 Sep 2024

#3. 'muscle tone'/exp 22,638 28 Sep 2024

#2. 'spasticity'/exp 30,068 28 Sep 2024

#1. 'moxibustion'/exp OR 'moxibustion' 17,196 28 Sep 2024

.......................................................

*S3*

Pubmed

(("moxibustion"[MeSH Terms] OR "moxibustion"[All Fields] OR ("moxibustion"[MeSH Terms] OR "moxibustion"[All Fields] OR "moxabustion"[All Fields])) AND ("stroke"[MeSH Terms] OR "stroke"[All Fields] OR "strokes"[All Fields] OR "stroke s"[All Fields] OR ("stroke"[MeSH Terms] OR "stroke"[All Fields] OR "strokes"[All Fields] OR "stroke s"[All Fields]) OR ("stroke"[MeSH Terms] OR "stroke"[All Fields] OR ("cerebrovascular"[All Fields] AND "accident"[All Fields]) OR "cerebrovascular accident"[All Fields]) OR ("stroke"[MeSH Terms] OR "stroke"[All Fields] OR ("cerebrovascular"[All Fields] AND "accidents"[All Fields]) OR "cerebrovascular accidents"[All Fields]) OR ("stroke"[MeSH Terms] OR "stroke"[All Fields] OR ("cerebral"[All Fields] AND "stroke"[All Fields]) OR "cerebral stroke"[All Fields]) OR ("stroke"[MeSH Terms] OR "stroke"[All Fields] OR ("cerebral"[All Fields] AND "strokes"[All Fields]) OR "cerebral strokes"[All Fields]) OR ("stroke"[MeSH Terms] OR "stroke"[All Fields] OR ("stroke"[All Fields] AND "cerebral"[All Fields]) OR "stroke cerebral"[All Fields]) OR ("stroke"[MeSH Terms] OR "stroke"[All Fields] OR ("strokes"[All Fields] AND "cerebral"[All Fields]) OR "strokes cerebral"[All Fields]) OR ("stroke"[MeSH Terms] OR "stroke"[All Fields] OR ("cerebrovascular"[All Fields] AND "apoplexy"[All Fields]) OR "cerebrovascular apoplexy"[All Fields]) OR ("stroke"[MeSH Terms] OR "stroke"[All Fields] OR ("apoplexy"[All Fields] AND "cerebrovascular"[All Fields]) OR "apoplexy cerebrovascular"[All Fields]) OR ("stroke"[MeSH Terms] OR "stroke"[All Fields] OR ("vascular"[All Fields] AND "accident"[All Fields] AND "brain"[All Fields]) OR "vascular accident brain"[All Fields]) OR ("stroke"[MeSH Terms] OR "stroke"[All Fields] OR ("brain"[All Fields] AND "vascular"[All Fields] AND "accident"[All Fields]) OR "brain vascular accident"[All Fields]) OR ("stroke"[MeSH Terms] OR "stroke"[All Fields] OR ("brain"[All Fields] AND "vascular"[All Fields] AND "accidents"[All Fields]) OR "brain vascular accidents"[All Fields]) OR ("stroke"[MeSH Terms] OR "stroke"[All Fields] OR ("vascular"[All Fields] AND "accidents"[All Fields] AND "brain"[All Fields])) OR ("stroke"[MeSH Terms] OR "stroke"[All Fields] OR ("cerebrovascular"[All Fields] AND "stroke"[All Fields]) OR "cerebrovascular stroke"[All Fields]) OR ("stroke"[MeSH Terms] OR "stroke"[All Fields] OR ("cerebrovascular"[All Fields] AND "strokes"[All Fields]) OR "cerebrovascular strokes"[All Fields]) OR ("stroke"[MeSH Terms] OR "stroke"[All Fields] OR ("stroke"[All Fields] AND "cerebrovascular"[All Fields]) OR "stroke cerebrovascular"[All Fields]) OR ("stroke"[MeSH Terms] OR "stroke"[All Fields] OR ("strokes"[All Fields] AND "cerebrovascular"[All Fields]) OR "strokes cerebrovascular"[All Fields]) OR ("apoplexies"[All Fields] OR "stroke"[MeSH Terms] OR "stroke"[All Fields] OR "apoplexy"[All Fields]) OR (("stroke"[MeSH Terms] OR "stroke"[All Fields] OR "cva"[All Fields]) AND ("stroke"[MeSH Terms] OR "stroke"[All Fields] OR ("cerebrovascular"[All Fields] AND "accident"[All Fields]) OR "cerebrovascular accident"[All Fields])) OR ("CVAs"[All Fields] AND ("stroke"[MeSH Terms] OR "stroke"[All Fields] OR ("cerebrovascular"[All Fields] AND "accident"[All Fields]) OR "cerebrovascular accident"[All Fields])) OR ("stroke"[MeSH Terms] OR "stroke"[All Fields] OR ("stroke"[All Fields] AND "acute"[All Fields]) OR "stroke acute"[All Fields]) OR ("stroke"[MeSH Terms] OR "stroke"[All Fields] OR ("acute"[All Fields] AND "stroke"[All Fields]) OR "acute stroke"[All Fields]) OR ("stroke"[MeSH Terms] OR "stroke"[All Fields] OR ("acute"[All Fields] AND "strokes"[All Fields]) OR "acute strokes"[All Fields]) OR ("stroke"[MeSH Terms] OR "stroke"[All Fields] OR ("strokes"[All Fields] AND "acute"[All Fields]) OR "strokes acute"[All Fields]) OR ("stroke"[MeSH Terms] OR "stroke"[All Fields] OR ("cerebrovascular"[All Fields] AND "accident"[All Fields] AND "acute"[All Fields]) OR "cerebrovascular accident acute"[All Fields]) OR ("stroke"[MeSH Terms] OR "stroke"[All Fields] OR ("acute"[All Fields] AND "cerebrovascular"[All Fields] AND "accident"[All Fields]) OR "acute cerebrovascular accident"[All Fields]) OR ("stroke"[MeSH Terms] OR "stroke"[All Fields] OR ("acute"[All Fields] AND "cerebrovascular"[All Fields] AND "accidents"[All Fields]) OR "acute cerebrovascular accidents"[All Fields]) OR ("stroke"[MeSH Terms] OR "stroke"[All Fields] OR ("cerebrovascular"[All Fields] AND "accidents"[All Fields] AND "acute"[All Fields]) OR "cerebrovascular accidents acute"[All Fields]) OR "Apoplexia"[All Fields] OR (("paralytic"[All Fields] OR "paralytical"[All Fields] OR "paralytics"[All Fields]) AND ("stroke"[MeSH Terms] OR "stroke"[All Fields] OR "strokes"[All Fields] OR "stroke s"[All Fields])) OR ("wind-stroke"[All Fields] AND ("syndrom"[All Fields] OR "syndromal"[All Fields] OR "syndromally"[All Fields] OR "syndrome"[MeSH Terms] OR "syndrome"[All Fields] OR "syndromes"[All Fields] OR "syndrome s"[All Fields] OR "syndromic"[All Fields] OR "syndroms"[All Fields])) OR ("cerebral haemorrhage"[All Fields] OR "cerebral hemorrhage"[MeSH Terms] OR ("cerebral"[All Fields] AND "hemorrhage"[All Fields]) OR "cerebral hemorrhage"[All Fields] OR ("cerebral hemorrhage"[MeSH Terms] OR ("cerebral"[All Fields] AND "hemorrhage"[All Fields]) OR "cerebral hemorrhage"[All Fields] OR ("hemorrhage"[All Fields] AND "cerebral"[All Fields]) OR "hemorrhage cerebral"[All Fields]) OR ("cerebral haemorrhages"[All Fields] OR "cerebral hemorrhage"[MeSH Terms] OR ("cerebral"[All Fields] AND "hemorrhage"[All Fields]) OR "cerebral hemorrhage"[All Fields] OR ("cerebral"[All Fields] AND "hemorrhages"[All Fields]) OR "cerebral hemorrhages"[All Fields]) OR ("cerebral hemorrhage"[MeSH Terms] OR ("cerebral"[All Fields] AND "hemorrhage"[All Fields]) OR "cerebral hemorrhage"[All Fields] OR ("hemorrhages"[All Fields] AND "cerebral"[All Fields]) OR "hemorrhages cerebral"[All Fields]) OR ("intracerebral haemorrhage"[All Fields] OR "cerebral hemorrhage"[MeSH Terms] OR ("cerebral"[All Fields] AND "hemorrhage"[All Fields]) OR "cerebral hemorrhage"[All Fields] OR ("intracerebral"[All Fields] AND "hemorrhage"[All Fields]) OR "intracerebral hemorrhage"[All Fields]) OR ("cerebral hemorrhage"[MeSH Terms] OR ("cerebral"[All Fields] AND "hemorrhage"[All Fields]) OR "cerebral hemorrhage"[All Fields] OR ("hemorrhage"[All Fields] AND "intracerebral"[All Fields]) OR "hemorrhage intracerebral"[All Fields]) OR ("cerebral hemorrhage"[MeSH Terms] OR ("cerebral"[All Fields] AND "hemorrhage"[All Fields]) OR "cerebral hemorrhage"[All Fields] OR ("hemorrhages"[All Fields] AND "intracerebral"[All Fields]) OR "hemorrhages intracerebral"[All Fields]) OR ("intracerebral haemorrhages"[All Fields] OR "cerebral hemorrhage"[MeSH Terms] OR ("cerebral"[All Fields] AND "hemorrhage"[All Fields]) OR "cerebral hemorrhage"[All Fields] OR ("intracerebral"[All Fields] AND "hemorrhages"[All Fields]) OR "intracerebral hemorrhages"[All Fields]) OR ("cerebral hemorrhage"[MeSH Terms] OR ("cerebral"[All Fields] AND "hemorrhage"[All Fields]) OR "cerebral hemorrhage"[All Fields] OR ("hemorrhage"[All Fields] AND "cerebrum"[All Fields])) OR ("cerebral hemorrhage"[MeSH Terms] OR ("cerebral"[All Fields] AND "hemorrhage"[All Fields]) OR "cerebral hemorrhage"[All Fields] OR ("cerebrum"[All Fields] AND "hemorrhage"[All Fields])) OR ("cerebral hemorrhage"[MeSH Terms] OR ("cerebral"[All Fields] AND "hemorrhage"[All Fields]) OR "cerebral hemorrhage"[All Fields] OR ("cerebrum"[All Fields] AND "hemorrhages"[All Fields])) OR ("cerebral hemorrhage"[MeSH Terms] OR ("cerebral"[All Fields] AND "hemorrhage"[All Fields]) OR "cerebral hemorrhage"[All Fields] OR ("hemorrhages"[All Fields] AND "cerebrum"[All Fields])) OR ("cerebral hemorrhage"[MeSH Terms] OR ("cerebral"[All Fields] AND "hemorrhage"[All Fields]) OR "cerebral hemorrhage"[All Fields] OR ("brain"[All Fields] AND "hemorrhage"[All Fields] AND "cerebral"[All Fields]) OR "brain hemorrhage cerebral"[All Fields]) OR ("cerebral hemorrhage"[MeSH Terms] OR ("cerebral"[All Fields] AND "hemorrhage"[All Fields]) OR "cerebral hemorrhage"[All Fields] OR ("brain"[All Fields] AND "hemorrhages"[All Fields] AND "cerebral"[All Fields])) OR ("cerebral hemorrhage"[MeSH Terms] OR ("cerebral"[All Fields] AND "hemorrhage"[All Fields]) OR "cerebral hemorrhage"[All Fields] OR ("cerebral"[All Fields] AND "brain"[All Fields] AND "hemorrhage"[All Fields]) OR "cerebral brain hemorrhage"[All Fields]) OR ("cerebral hemorrhage"[MeSH Terms] OR ("cerebral"[All Fields] AND "hemorrhage"[All Fields]) OR "cerebral hemorrhage"[All Fields] OR ("cerebral"[All Fields] AND "brain"[All Fields] AND "hemorrhages"[All Fields])) OR ("cerebral hemorrhage"[MeSH Terms] OR ("cerebral"[All Fields] AND "hemorrhage"[All Fields]) OR "cerebral hemorrhage"[All Fields] OR ("hemorrhage"[All Fields] AND "cerebral"[All Fields] AND "brain"[All Fields])) OR ("cerebral hemorrhage"[MeSH Terms] OR ("cerebral"[All Fields] AND "hemorrhage"[All Fields]) OR "cerebral hemorrhage"[All Fields] OR ("hemorrhages"[All Fields] AND "cerebral"[All Fields] AND "brain"[All Fields])) OR ("cerebral parenchymal haemorrhage"[All Fields] OR "cerebral hemorrhage"[MeSH Terms] OR ("cerebral"[All Fields] AND "hemorrhage"[All Fields]) OR "cerebral hemorrhage"[All Fields] OR ("cerebral"[All Fields] AND "parenchymal"[All Fields] AND "hemorrhage"[All Fields]) OR "cerebral parenchymal hemorrhage"[All Fields]) OR ("cerebral hemorrhage"[MeSH Terms] OR ("cerebral"[All Fields] AND "hemorrhage"[All Fields]) OR "cerebral hemorrhage"[All Fields] OR ("cerebral"[All Fields] AND "parenchymal"[All Fields] AND "hemorrhages"[All Fields]) OR "cerebral parenchymal hemorrhages"[All Fields]) OR ("cerebral hemorrhage"[MeSH Terms] OR ("cerebral"[All Fields] AND "hemorrhage"[All Fields]) OR "cerebral hemorrhage"[All Fields] OR ("hemorrhage"[All Fields] AND "cerebral"[All Fields] AND "parenchymal"[All Fields])) OR ("cerebral hemorrhage"[MeSH Terms] OR ("cerebral"[All Fields] AND "hemorrhage"[All Fields]) OR "cerebral hemorrhage"[All Fields] OR ("hemorrhages"[All Fields] AND "cerebral"[All Fields] AND "parenchymal"[All Fields])) OR ("cerebral hemorrhage"[MeSH Terms] OR ("cerebral"[All Fields] AND "hemorrhage"[All Fields]) OR "cerebral hemorrhage"[All Fields] OR ("parenchymal"[All Fields] AND "hemorrhage"[All Fields] AND "cerebral"[All Fields]) OR "parenchymal hemorrhage cerebral"[All Fields]) OR ("cerebral hemorrhage"[MeSH Terms] OR ("cerebral"[All Fields] AND "hemorrhage"[All Fields]) OR "cerebral hemorrhage"[All Fields] OR ("parenchymal"[All Fields] AND "hemorrhages"[All Fields] AND "cerebral"[All Fields])) OR ("cerebral haemorrhage"[All Fields] OR "cerebral hemorrhage"[MeSH Terms] OR ("cerebral"[All Fields] AND "hemorrhage"[All Fields]) OR "cerebral hemorrhage"[All Fields]) OR (("blood"[MeSH Subheading] OR "blood"[All Fields] OR "blood"[MeSH Terms] OR "bloods"[All Fields] OR "haematology"[All Fields] OR "hematology"[MeSH Terms] OR "hematology"[All Fields] OR "haematoma"[All Fields] OR "hematoma"[MeSH Terms] OR "hematoma"[All Fields] OR "haemorrhage"[All Fields] OR "hemorrhage"[MeSH Terms] OR "hemorrhage"[All Fields] OR "haemorrhages"[All Fields] OR "hemorrhages"[All Fields] OR "haemorrhagic"[All Fields] OR "haemorrhaging"[All Fields] OR "hematologies"[All Fields] OR "haematomas"[All Fields] OR "hematomas"[All Fields] OR "hematoma s"[All Fields] OR "hematomae"[All Fields] OR "hemorrhaged"[All Fields] OR "hemorrhagic"[All Fields] OR "hemorrhagical"[All Fields] OR "hemorrhaging"[All Fields]) AND ("brain"[MeSH Terms] OR "brain"[All Fields] OR "brains"[All Fields] OR "brain s"[All Fields]))) OR ("cerebral infarction"[MeSH Terms] OR ("cerebral"[All Fields] AND "infarction"[All Fields]) OR "cerebral infarction"[All Fields] OR ("cerebral infarction"[MeSH Terms] OR ("cerebral"[All Fields] AND "infarction"[All Fields]) OR "cerebral infarction"[All Fields] OR ("cerebral"[All Fields] AND "infarctions"[All Fields]) OR "cerebral infarctions"[All Fields]) OR ("cerebral infarction"[MeSH Terms] OR ("cerebral"[All Fields] AND "infarction"[All Fields]) OR "cerebral infarction"[All Fields] OR ("infarctions"[All Fields] AND "cerebral"[All Fields]) OR "infarctions cerebral"[All Fields]) OR ("cerebral infarction"[MeSH Terms] OR ("cerebral"[All Fields] AND "infarction"[All Fields]) OR "cerebral infarction"[All Fields] OR ("cerebral"[All Fields] AND "infarct"[All Fields]) OR "cerebral infarct"[All Fields]) OR ("cerebral infarction"[MeSH Terms] OR ("cerebral"[All Fields] AND "infarction"[All Fields]) OR "cerebral infarction"[All Fields] OR ("cerebral"[All Fields] AND "infarcts"[All Fields]) OR "cerebral infarcts"[All Fields]) OR ("cerebral infarction"[MeSH Terms] OR ("cerebral"[All Fields] AND "infarction"[All Fields]) OR "cerebral infarction"[All Fields] OR ("infarct"[All Fields] AND "cerebral"[All Fields]) OR "infarct cerebral"[All Fields]) OR ("cerebral infarction"[MeSH Terms] OR ("cerebral"[All Fields] AND "infarction"[All Fields]) OR "cerebral infarction"[All Fields] OR ("infarcts"[All Fields] AND "cerebral"[All Fields]) OR "infarcts cerebral"[All Fields]) OR ("cerebral infarction"[MeSH Terms] OR ("cerebral"[All Fields] AND "infarction"[All Fields]) OR "cerebral infarction"[All Fields] OR ("infarction"[All Fields] AND "cerebral"[All Fields]) OR "infarction cerebral"[All Fields]) OR ("cerebral infarction"[MeSH Terms] OR ("cerebral"[All Fields] AND "infarction"[All Fields]) OR "cerebral infarction"[All Fields] OR ("anterior"[All Fields] AND "choroidal"[All Fields] AND "artery"[All Fields] AND "infarction"[All Fields]) OR "anterior choroidal artery infarction"[All Fields]) OR ("cerebral infarction"[MeSH Terms] OR ("cerebral"[All Fields] AND "infarction"[All Fields]) OR "cerebral infarction"[All Fields] OR ("posterior"[All Fields] AND "choroidal"[All Fields] AND "artery"[All Fields] AND "infarction"[All Fields]) OR "posterior choroidal artery infarction"[All Fields]) OR ("cerebral infarction"[MeSH Terms] OR ("cerebral"[All Fields] AND "infarction"[All Fields]) OR "cerebral infarction"[All Fields] OR ("subcortical"[All Fields] AND "infarction"[All Fields]) OR "subcortical infarction"[All Fields]) OR ("cerebral infarction"[MeSH Terms] OR ("cerebral"[All Fields] AND "infarction"[All Fields]) OR "cerebral infarction"[All Fields] OR ("infarctions"[All Fields] AND "subcortical"[All Fields]) OR "infarctions subcortical"[All Fields]) OR ("cerebral infarction"[MeSH Terms] OR ("cerebral"[All Fields] AND "infarction"[All Fields]) OR "cerebral infarction"[All Fields] OR ("infarction"[All Fields] AND "subcortical"[All Fields]) OR "infarction subcortical"[All Fields]) OR ("cerebral infarction"[MeSH Terms] OR ("cerebral"[All Fields] AND "infarction"[All Fields]) OR "cerebral infarction"[All Fields] OR ("subcortical"[All Fields] AND "infarctions"[All Fields]) OR "subcortical infarctions"[All Fields]) OR ("cerebral infarction"[MeSH Terms] OR ("cerebral"[All Fields] AND "infarction"[All Fields]) OR "cerebral infarction"[All Fields] OR ("cerebral"[All Fields] AND "infarction"[All Fields] AND "left"[All Fields] AND "hemisphere"[All Fields])) OR ("cerebral infarction"[MeSH Terms] OR ("cerebral"[All Fields] AND "infarction"[All Fields]) OR "cerebral infarction"[All Fields] OR ("cerebral"[All Fields] AND "left"[All Fields] AND "hemisphere"[All Fields] AND "infarction"[All Fields])) OR ("cerebral infarction"[MeSH Terms] OR ("cerebral"[All Fields] AND "infarction"[All Fields]) OR "cerebral infarction"[All Fields] OR ("infarction"[All Fields] AND "cerebral"[All Fields] AND "left"[All Fields] AND "hemisphere"[All Fields])) OR ("cerebral infarction"[MeSH Terms] OR ("cerebral"[All Fields] AND "infarction"[All Fields]) OR "cerebral infarction"[All Fields] OR ("left"[All Fields] AND "hemisphere"[All Fields] AND "infarction"[All Fields] AND "cerebral"[All Fields])) OR ("cerebral infarction"[MeSH Terms] OR ("cerebral"[All Fields] AND "infarction"[All Fields]) OR "cerebral infarction"[All Fields] OR ("infarction"[All Fields] AND "left"[All Fields] AND "hemisphere"[All Fields] AND "cerebral"[All Fields])) OR ("cerebral infarction"[MeSH Terms] OR ("cerebral"[All Fields] AND "infarction"[All Fields]) OR "cerebral infarction"[All Fields] OR ("left"[All Fields] AND "hemisphere"[All Fields] AND "cerebral"[All Fields] AND "infarction"[All Fields]) OR "left hemisphere cerebral infarction"[All Fields]) OR ("cerebral infarction"[MeSH Terms] OR ("cerebral"[All Fields] AND "infarction"[All Fields]) OR "cerebral infarction"[All Fields] OR ("cerebral"[All Fields] AND "infarction"[All Fields] AND "right"[All Fields] AND "hemisphere"[All Fields])) OR ("cerebral infarction"[MeSH Terms] OR ("cerebral"[All Fields] AND "infarction"[All Fields]) OR "cerebral infarction"[All Fields] OR ("cerebral"[All Fields] AND "right"[All Fields] AND "hemisphere"[All Fields] AND "infarction"[All Fields])) OR ("cerebral infarction"[MeSH Terms] OR ("cerebral"[All Fields] AND "infarction"[All Fields]) OR "cerebral infarction"[All Fields] OR ("infarction"[All Fields] AND "cerebral"[All Fields] AND "right"[All Fields] AND "hemisphere"[All Fields])) OR ("cerebral infarction"[MeSH Terms] OR ("cerebral"[All Fields] AND "infarction"[All Fields]) OR "cerebral infarction"[All Fields] OR ("infarction"[All Fields] AND "right"[All Fields] AND "hemisphere"[All Fields] AND "cerebral"[All Fields])) OR ("cerebral infarction"[MeSH Terms] OR ("cerebral"[All Fields] AND "infarction"[All Fields]) OR "cerebral infarction"[All Fields] OR ("right"[All Fields] AND "hemisphere"[All Fields] AND "infarction"[All Fields] AND "cerebral"[All Fields])) OR ("cerebral infarction"[MeSH Terms] OR ("cerebral"[All Fields] AND "infarction"[All Fields]) OR "cerebral infarction"[All Fields] OR ("right"[All Fields] AND "hemisphere"[All Fields] AND "cerebral"[All Fields] AND "infarction"[All Fields]) OR "right hemisphere cerebral infarction"[All Fields])) OR ("brain infarction"[MeSH Terms] OR ("brain"[All Fields] AND "infarction"[All Fields]) OR "brain infarction"[All Fields] OR ("brain infarction"[MeSH Terms] OR ("brain"[All Fields] AND "infarction"[All Fields]) OR "brain infarction"[All Fields] OR ("brain"[All Fields] AND "infarctions"[All Fields]) OR "brain infarctions"[All Fields]) OR ("brain infarction"[MeSH Terms] OR ("brain"[All Fields] AND "infarction"[All Fields]) OR "brain infarction"[All Fields] OR ("infarction"[All Fields] AND "brain"[All Fields]) OR "infarction brain"[All Fields]) OR ("brain infarction"[MeSH Terms] OR ("brain"[All Fields] AND "infarction"[All Fields]) OR "brain infarction"[All Fields] OR ("infarctions"[All Fields] AND "brain"[All Fields]) OR "infarctions brain"[All Fields]) OR ("brain infarction"[MeSH Terms] OR ("brain"[All Fields] AND "infarction"[All Fields]) OR "brain infarction"[All Fields] OR ("brain"[All Fields] AND "infarct"[All Fields]) OR "brain infarct"[All Fields]) OR ("brain infarction"[MeSH Terms] OR ("brain"[All Fields] AND "infarction"[All Fields]) OR "brain infarction"[All Fields] OR ("brain"[All Fields] AND "infarcts"[All Fields]) OR "brain infarcts"[All Fields]) OR ("brain infarction"[MeSH Terms] OR ("brain"[All Fields] AND "infarction"[All Fields]) OR "brain infarction"[All Fields] OR ("infarct"[All Fields] AND "brain"[All Fields]) OR "infarct brain"[All Fields]) OR ("brain infarction"[MeSH Terms] OR ("brain"[All Fields] AND "infarction"[All Fields]) OR "brain infarction"[All Fields] OR ("infarcts"[All Fields] AND "brain"[All Fields]) OR "infarcts brain"[All Fields]) OR ("brain infarction"[MeSH Terms] OR ("brain"[All Fields] AND "infarction"[All Fields]) OR "brain infarction"[All Fields] OR ("venous"[All Fields] AND "infarction"[All Fields] AND "brain"[All Fields]) OR "venous infarction brain"[All Fields]) OR ("brain infarction"[MeSH Terms] OR ("brain"[All Fields] AND "infarction"[All Fields]) OR "brain infarction"[All Fields] OR ("brain"[All Fields] AND "venous"[All Fields] AND "infarction"[All Fields]) OR "brain venous infarction"[All Fields]) OR ("brain infarction"[MeSH Terms] OR ("brain"[All Fields] AND "infarction"[All Fields]) OR "brain infarction"[All Fields] OR ("brain"[All Fields] AND "venous"[All Fields] AND "infarctions"[All Fields])) OR ("brain infarction"[MeSH Terms] OR ("brain"[All Fields] AND "infarction"[All Fields]) OR "brain infarction"[All Fields] OR ("infarction"[All Fields] AND "brain"[All Fields] AND "venous"[All Fields])) OR ("brain infarction"[MeSH Terms] OR ("brain"[All Fields] AND "infarction"[All Fields]) OR "brain infarction"[All Fields] OR ("infarctions"[All Fields] AND "brain"[All Fields] AND "venous"[All Fields])) OR ("brain infarction"[MeSH Terms] OR ("brain"[All Fields] AND "infarction"[All Fields]) OR "brain infarction"[All Fields] OR ("venous"[All Fields] AND "infarctions"[All Fields] AND "brain"[All Fields]) OR "venous infarctions brain"[All Fields]) OR ("brain infarction"[MeSH Terms] OR ("brain"[All Fields] AND "infarction"[All Fields]) OR "brain infarction"[All Fields] OR ("brain"[All Fields] AND "infarction"[All Fields] AND "venous"[All Fields])) OR ("brain infarction"[MeSH Terms] OR ("brain"[All Fields] AND "infarction"[All Fields]) OR "brain infarction"[All Fields] OR ("brain"[All Fields] AND "infarctions"[All Fields] AND "venous"[All Fields])) OR ("brain infarction"[MeSH Terms] OR ("brain"[All Fields] AND "infarction"[All Fields]) OR "brain infarction"[All Fields] OR ("infarctions"[All Fields] AND "venous"[All Fields] AND "brain"[All Fields])) OR ("brain infarction"[MeSH Terms] OR ("brain"[All Fields] AND "infarction"[All Fields]) OR "brain infarction"[All Fields] OR ("infarction"[All Fields] AND "venous"[All Fields] AND "brain"[All Fields])) OR ("brain infarction"[MeSH Terms] OR ("brain"[All Fields] AND "infarction"[All Fields]) OR "brain infarction"[All Fields] OR ("venous"[All Fields] AND "brain"[All Fields] AND "infarction"[All Fields])) OR ("brain infarction"[MeSH Terms] OR ("brain"[All Fields] AND "infarction"[All Fields]) OR "brain infarction"[All Fields] OR ("venous"[All Fields] AND "brain"[All Fields] AND "infarctions"[All Fields]) OR "venous brain infarctions"[All Fields]) OR ("brain infarction"[MeSH Terms] OR ("brain"[All Fields] AND "infarction"[All Fields]) OR "brain infarction"[All Fields] OR ("anterior"[All Fields] AND "cerebral"[All Fields] AND "circulation"[All Fields] AND "infarction"[All Fields]) OR "anterior cerebral circulation infarction"[All Fields]) OR ("brain infarction"[MeSH Terms] OR ("brain"[All Fields] AND "infarction"[All Fields]) OR "brain infarction"[All Fields] OR ("infarction"[All Fields] AND "anterior"[All Fields] AND "cerebral"[All Fields] AND "circulation"[All Fields])) OR ("brain infarction"[MeSH Terms] OR ("brain"[All Fields] AND "infarction"[All Fields]) OR "brain infarction"[All Fields] OR ("anterior"[All Fields] AND "circulation"[All Fields] AND "brain"[All Fields] AND "infarction"[All Fields]) OR "anterior circulation brain infarction"[All Fields]) OR ("brain infarction"[MeSH Terms] OR ("brain"[All Fields] AND "infarction"[All Fields]) OR "brain infarction"[All Fields] OR ("anterior"[All Fields] AND "circulation"[All Fields] AND "infarction"[All Fields] AND "brain"[All Fields])) OR ("brain infarction"[MeSH Terms] OR ("brain"[All Fields] AND "infarction"[All Fields]) OR "brain infarction"[All Fields] OR ("brain"[All Fields] AND "infarction"[All Fields] AND "anterior"[All Fields] AND "circulation"[All Fields])) OR ("brain infarction"[MeSH Terms] OR ("brain"[All Fields] AND "infarction"[All Fields]) OR "brain infarction"[All Fields] OR ("infarction"[All Fields] AND "anterior"[All Fields] AND "circulation"[All Fields] AND "brain"[All Fields])) OR ("brain infarction"[MeSH Terms] OR ("brain"[All Fields] AND "infarction"[All Fields]) OR "brain infarction"[All Fields] OR ("infarction"[All Fields] AND "brain"[All Fields] AND "anterior"[All Fields] AND "circulation"[All Fields])) OR ("brain infarction"[MeSH Terms] OR ("brain"[All Fields] AND "infarction"[All Fields]) OR "brain infarction"[All Fields] OR ("brain"[All Fields] AND "infarction"[All Fields] AND "posterior"[All Fields] AND "circulation"[All Fields]) OR "brain infarction posterior circulation"[All Fields]) OR ("brain infarction"[MeSH Terms] OR ("brain"[All Fields] AND "infarction"[All Fields]) OR "brain infarction"[All Fields] OR ("posterior"[All Fields] AND "circulation"[All Fields] AND "brain"[All Fields] AND "infarction"[All Fields]) OR "posterior circulation brain infarction"[All Fields]) OR ("brain infarction"[MeSH Terms] OR ("brain"[All Fields] AND "infarction"[All Fields]) OR "brain infarction"[All Fields] OR ("posterior"[All Fields] AND "circulation"[All Fields] AND "infarction"[All Fields] AND "brain"[All Fields])) OR ("brain infarction"[MeSH Terms] OR ("brain"[All Fields] AND "infarction"[All Fields]) OR "brain infarction"[All Fields] OR ("infarction"[All Fields] AND "brain"[All Fields] AND "posterior"[All Fields] AND "circulation"[All Fields])) OR ("brain infarction"[MeSH Terms] OR ("brain"[All Fields] AND "infarction"[All Fields]) OR "brain infarction"[All Fields] OR ("infarction"[All Fields] AND "posterior"[All Fields] AND "circulation"[All Fields] AND "brain"[All Fields])))) AND ("muscle tonus"[MeSH Terms] OR ("muscle"[All Fields] AND "tonus"[All Fields]) OR "muscle tonus"[All Fields] OR ("muscle tonus"[MeSH Terms] OR ("muscle"[All Fields] AND "tonus"[All Fields]) OR "muscle tonus"[All Fields] OR ("tonus"[All Fields] AND "muscle"[All Fields]) OR "tonus muscle"[All Fields]) OR ("muscle tonus"[MeSH Terms] OR ("muscle"[All Fields] AND "tonus"[All Fields]) OR "muscle tonus"[All Fields] OR ("muscle"[All Fields] AND "tension"[All Fields]) OR "muscle tension"[All Fields]) OR ("muscle tonus"[MeSH Terms] OR ("muscle"[All Fields] AND "tonus"[All Fields]) OR "muscle tonus"[All Fields] OR ("tension"[All Fields] AND "muscle"[All Fields]) OR "tension muscle"[All Fields]) OR ("muscle tonus"[MeSH Terms] OR ("muscle"[All Fields] AND "tonus"[All Fields]) OR "muscle tonus"[All Fields] OR ("muscular"[All Fields] AND "tension"[All Fields]) OR "muscular tension"[All Fields]) OR ("muscle tonus"[MeSH Terms] OR ("muscle"[All Fields] AND "tonus"[All Fields]) OR "muscle tonus"[All Fields] OR ("tension"[All Fields] AND "muscular"[All Fields]) OR "tension muscular"[All Fields]) OR ("muscle tonus"[MeSH Terms] OR ("muscle"[All Fields] AND "tonus"[All Fields]) OR "muscle tonus"[All Fields] OR ("muscle"[All Fields] AND "tightness"[All Fields]) OR "muscle tightness"[All Fields]) OR ("muscle tonus"[MeSH Terms] OR ("muscle"[All Fields] AND "tonus"[All Fields]) OR "muscle tonus"[All Fields] OR ("tightness"[All Fields] AND "muscle"[All Fields]) OR "tightness muscle"[All Fields]) OR ("hemiplegia"[MeSH Terms] OR "hemiplegia"[All Fields] OR "hemiplegias"[All Fields] OR ("hemiplegia"[MeSH Terms] OR "hemiplegia"[All Fields] OR ("hemiplegia"[All Fields] AND "spastic"[All Fields]) OR "hemiplegia spastic"[All Fields]) OR ("hemiplegia"[MeSH Terms] OR "hemiplegia"[All Fields] OR ("hemiplegias"[All Fields] AND "spastic"[All Fields])) OR ("hemiplegia"[MeSH Terms] OR "hemiplegia"[All Fields] OR ("spastic"[All Fields] AND "hemiplegia"[All Fields]) OR "spastic hemiplegia"[All Fields]) OR ("hemiplegia"[MeSH Terms] OR "hemiplegia"[All Fields] OR ("spastic"[All Fields] AND "hemiplegias"[All Fields]) OR "spastic hemiplegias"[All Fields])) OR ("paralysing"[All Fields] OR "paralysis"[MeSH Terms] OR "paralysis"[All Fields] OR "paralyse"[All Fields] OR "paralysed"[All Fields] OR "paralyses"[All Fields] OR ("paralysing"[All Fields] OR "paralysis"[MeSH Terms] OR "paralysis"[All Fields] OR "paralyse"[All Fields] OR "paralysed"[All Fields] OR "paralyses"[All Fields]) OR ("paralysis"[MeSH Terms] OR "paralysis"[All Fields] OR "palsied"[All Fields] OR "palsies"[All Fields] OR "palsy"[All Fields]) OR ("paralysis"[MeSH Terms] OR "paralysis"[All Fields] OR "palsied"[All Fields] OR "palsies"[All Fields] OR "palsy"[All Fields]) OR ("paralysis"[MeSH Terms] OR "paralysis"[All Fields] OR "plegia"[All Fields] OR "plegias"[All Fields]) OR ("paralysis"[MeSH Terms] OR "paralysis"[All Fields] OR "plegia"[All Fields] OR "plegias"[All Fields]) OR ("paralysis"[MeSH Terms] OR "paralysis"[All Fields] OR ("todd"[All Fields] AND "paralysis"[All Fields]) OR "todd paralysis"[All Fields]) OR ("paralysis"[MeSH Terms] OR "paralysis"[All Fields] OR ("paralysis"[All Fields] AND "todd"[All Fields]) OR "paralysis todd"[All Fields]) OR ("paralysis"[MeSH Terms] OR "paralysis"[All Fields] OR ("todd s"[All Fields] AND "paralysis"[All Fields]) OR "todd s paralysis"[All Fields]) OR ("paralysis"[MeSH Terms] OR "paralysis"[All Fields] OR ("paralysis"[All Fields] AND "todd s"[All Fields]) OR "paralysis todd s"[All Fields]) OR ("paralysis"[MeSH Terms] OR "paralysis"[All Fields] OR ("todds"[All Fields] AND "paralysis"[All Fields]))) OR ("muscle spasticity"[MeSH Terms] OR ("muscle"[All Fields] AND "spasticity"[All Fields]) OR "muscle spasticity"[All Fields] OR ("muscle spasticity"[MeSH Terms] OR ("muscle"[All Fields] AND "spasticity"[All Fields]) OR "muscle spasticity"[All Fields] OR ("spasticity"[All Fields] AND "muscle"[All Fields]) OR "spasticity muscle"[All Fields]) OR ("muscle spasticity"[MeSH Terms] OR ("muscle"[All Fields] AND "spasticity"[All Fields]) OR "muscle spasticity"[All Fields] OR ("clasp"[All Fields] AND "knife"[All Fields] AND "spasticity"[All Fields]) OR "clasp knife spasticity"[All Fields]) OR ("muscle spasticity"[MeSH Terms] OR ("muscle"[All Fields] AND "spasticity"[All Fields]) OR "muscle spasticity"[All Fields] OR ("clasp"[All Fields] AND "knife"[All Fields] AND "spasticity"[All Fields]) OR "clasp knife spasticity"[All Fields]) OR (("muscle spasticity"[MeSH Terms] OR ("muscle"[All Fields] AND "spasticity"[All Fields]) OR "muscle spasticity"[All Fields] OR "spastic"[All Fields] OR "spasticity"[All Fields] OR "spastics"[All Fields] OR "spasticities"[All Fields]) AND "Clasp-Knife"[All Fields]) OR ("muscle spasticity"[MeSH Terms] OR ("muscle"[All Fields] AND "spasticity"[All Fields]) OR "muscle spasticity"[All Fields] OR "spastic"[All Fields] OR "spasticity"[All Fields] OR "spastics"[All Fields] OR "spasticities"[All Fields])) OR ("spasm"[MeSH Terms] OR "spasm"[All Fields] OR "spasms"[All Fields] OR ("spasm"[MeSH Terms] OR "spasm"[All Fields] OR "spasms"[All Fields]) OR ("spasm"[MeSH Terms] OR "spasm"[All Fields] OR ("muscle"[All Fields] AND "spasm"[All Fields]) OR "muscle spasm"[All Fields]) OR ("spasm"[MeSH Terms] OR "spasm"[All Fields] OR ("muscle"[All Fields] AND "spasms"[All Fields]) OR "muscle spasms"[All Fields]) OR ("spasm"[MeSH Terms] OR "spasm"[All Fields] OR ("spasm"[All Fields] AND "muscle"[All Fields]) OR "spasm muscle"[All Fields]) OR ("spasm"[MeSH Terms] OR "spasm"[All Fields] OR ("spasms"[All Fields] AND "muscle"[All Fields]) OR "spasms muscle"[All Fields]) OR ("spasm"[MeSH Terms] OR "spasm"[All Fields] OR ("muscular"[All Fields] AND "spasm"[All Fields]) OR "muscular spasm"[All Fields]) OR ("spasm"[MeSH Terms] OR "spasm"[All Fields] OR ("muscular"[All Fields] AND "spasms"[All Fields]) OR "muscular spasms"[All Fields]) OR ("spasm"[MeSH Terms] OR "spasm"[All Fields] OR ("spasm"[All Fields] AND "muscular"[All Fields]) OR "spasm muscular"[All Fields]) OR ("spasm"[MeSH Terms] OR "spasm"[All Fields] OR ("spasms"[All Fields] AND "muscular"[All Fields]) OR "spasms muscular"[All Fields]) OR ("spasm"[MeSH Terms] OR "spasm"[All Fields] OR ("spasm"[All Fields] AND "ciliary"[All Fields] AND "body"[All Fields])) OR ("spasm"[MeSH Terms] OR "spasm"[All Fields] OR ("ciliary"[All Fields] AND "body"[All Fields] AND "spasm"[All Fields])) OR ("spasm"[MeSH Terms] OR "spasm"[All Fields] OR ("ciliary"[All Fields] AND "body"[All Fields] AND "spasms"[All Fields])) OR ("spasm"[MeSH Terms] OR "spasm"[All Fields] OR ("spasms"[All Fields] AND "ciliary"[All Fields] AND "body"[All Fields])) OR ("spasm"[MeSH Terms] OR "spasm"[All Fields] OR ("spasm"[All Fields] AND "generalized"[All Fields]) OR "spasm generalized"[All Fields]) OR ("generalised spasm"[All Fields] OR "spasm"[MeSH Terms] OR "spasm"[All Fields] OR ("generalized"[All Fields] AND "spasm"[All Fields]) OR "generalized spasm"[All Fields]) OR ("generalised spasms"[All Fields] OR "spasm"[MeSH Terms] OR "spasm"[All Fields] OR ("generalized"[All Fields] AND "spasms"[All Fields]) OR "generalized spasms"[All Fields]) OR ("spasm"[MeSH Terms] OR "spasm"[All Fields] OR ("spasms"[All Fields] AND "generalized"[All Fields]) OR "spasms generalized"[All Fields])) OR ("muscle cramp"[MeSH Terms] OR ("muscle"[All Fields] AND "cramp"[All Fields]) OR "muscle cramp"[All Fields] OR ("muscle cramp"[MeSH Terms] OR ("muscle"[All Fields] AND "cramp"[All Fields]) OR "muscle cramp"[All Fields] OR ("cramp"[All Fields] AND "muscle"[All Fields]) OR "cramp muscle"[All Fields]) OR ("muscle cramp"[MeSH Terms] OR ("muscle"[All Fields] AND "cramp"[All Fields]) OR "muscle cramp"[All Fields] OR ("cramps"[All Fields] AND "muscle"[All Fields]) OR "cramps muscle"[All Fields]) OR ("spasm"[MeSH Terms] OR "spasm"[All Fields] OR ("muscle"[All Fields] AND "cramps"[All Fields]) OR "muscle cramps"[All Fields] OR "muscle cramp"[MeSH Terms] OR ("muscle"[All Fields] AND "cramp"[All Fields]) OR "muscle cramp"[All Fields]) OR ("muscle cramp"[MeSH Terms] OR ("muscle"[All Fields] AND "cramp"[All Fields]) OR "muscle cramp"[All Fields] OR "cramp"[All Fields] OR "cramping"[All Fields] OR "cramps"[All Fields]) OR ("muscle cramp"[MeSH Terms] OR ("muscle"[All Fields] AND "cramp"[All Fields]) OR "muscle cramp"[All Fields] OR "cramp"[All Fields] OR "cramping"[All Fields] OR "cramps"[All Fields]) OR ("muscle cramp"[MeSH Terms] OR ("muscle"[All Fields] AND "cramp"[All Fields]) OR "muscle cramp"[All Fields] OR ("muscular"[All Fields] AND "cramp"[All Fields]) OR "muscular cramp"[All Fields]) OR ("muscle cramp"[MeSH Terms] OR ("muscle"[All Fields] AND "cramp"[All Fields]) OR "muscle cramp"[All Fields] OR ("cramp"[All Fields] AND "muscular"[All Fields]) OR "cramp muscular"[All Fields]) OR ("muscle cramp"[MeSH Terms] OR ("muscle"[All Fields] AND "cramp"[All Fields]) OR "muscle cramp"[All Fields] OR ("cramps"[All Fields] AND "muscular"[All Fields]) OR "cramps muscular"[All Fields]) OR ("muscle cramp"[MeSH Terms] OR ("muscle"[All Fields] AND "cramp"[All Fields]) OR "muscle cramp"[All Fields] OR ("muscular"[All Fields] AND "cramps"[All Fields]) OR "muscular cramps"[All Fields]) OR ("muscle cramp"[MeSH Terms] OR ("muscle"[All Fields] AND "cramp"[All Fields]) OR "muscle cramp"[All Fields] OR ("limb"[All Fields] AND "cramp"[All Fields]) OR "limb cramp"[All Fields]) OR ("muscle cramp"[MeSH Terms] OR ("muscle"[All Fields] AND "cramp"[All Fields]) OR "muscle cramp"[All Fields] OR ("cramp"[All Fields] AND "limb"[All Fields])) OR ("muscle cramp"[MeSH Terms] OR ("muscle"[All Fields] AND "cramp"[All Fields]) OR "muscle cramp"[All Fields] OR ("cramps"[All Fields] AND "limb"[All Fields])) OR ("muscle cramp"[MeSH Terms] OR ("muscle"[All Fields] AND "cramp"[All Fields]) OR "muscle cramp"[All Fields] OR ("limb"[All Fields] AND "cramps"[All Fields]) OR "limb cramps"[All Fields]) OR ("convulsants"[Pharmacological Action] OR "convulsants"[MeSH Terms] OR "convulsants"[All Fields] OR "convulsant"[All Fields] OR "convulse"[All Fields] OR "convulsed"[All Fields] OR "convulsing"[All Fields] OR "convulsive"[All Fields] OR "convulsives"[All Fields] OR "seizures"[MeSH Terms] OR "seizures"[All Fields] OR "convulsion"[All Fields] OR "convulsions"[All Fields]) OR ("twitch"[All Fields] OR "twitched"[All Fields] OR "twitches"[All Fields] OR "twitching"[All Fields] OR "twitchings"[All Fields]) OR ("seizures"[MeSH Terms] OR "seizures"[All Fields] OR "fit"[All Fields]) OR "jerk"[All Fields] OR "tetang"[All Fields]))) AND (randomizedcontrolledtrial[Filter])

*S4*

Web of science：10+35=45

35

<https://webofscience.clarivate.cn/wos/woscc/summary/e10af9d3-6219-4435-bdb5-2b34f8549ecd-010cdbf423/relevance/1>

10

<https://webofscience.clarivate.cn/wos/woscc/summary/fa84e0fb-83c3-4251-9690-0eb4f02087cf-0107531fe6/relevance/1>
